# Quantitative assessment of retinal vasculature changes in systemic lupus erythematosus using wide-field OCTA and the correlation with disease activity

**DOI:** 10.3389/fimmu.2024.1340224

**Published:** 2024-01-29

**Authors:** Lihui Meng, Lulu Chen, Chenxi Zhang, Huan Chen, Jingyuan Yang, Yuelin Wang, Wenfei Zhang, Shiyu Cheng, Qing Zhao, Xinyu Zhao, Youxin Chen

**Affiliations:** ^1^ Department of Ophthalmology, Peking Union Medical College Hospital, Chinese Academy of Medical Sciences, Beijing, China; ^2^ Key Laboratory of Ocular Fundus Diseases, Chinese Academy of Medical Sciences and Peking Union Medical College, Beijing, China

**Keywords:** prospective, cross-sectional, observational study, systemic lupus erythematosus, retinal vessel density, optical coherence tomography angiography, disease activity

## Abstract

**Purpose:**

To assess the retinal vasculature changes quantitatively using wide-field optical coherence tomography angiography (OCTA) in systemic lupus erythematosus (SLE), and explore its correlation with systemic clinical features.

**Design:**

Prospective, cross-sectional, observational study.

**Participants and controls:**

Patients with SLE who presented to the Ophthalmology Department of Peking Union Medical College Hospital from November 2022 to April 2023 were collected. The subjects were divided into retinopathy and without retinopathy groups. Age and gender-matched healthy subjects were selected as controls.

**Methods:**

Patients with SLE and control subjects were imaged with 24×20 mm OCTA scans centered on the fovea and 6×6 mm OCTA scans centered on the optic disc. The sub-layers of OCTA images were stratified by the built-in software of the device and then the retinal thickness and vessel density were measured automatically. The characteristics of retinal OCTA parameters of SLE and its correlation with systemic clinical indicators of patients without retinopathy were analyzed.

**Main outcome measures:**

OCTA parameters, visual acuity, intraocular pressure, and systemic clinical indicators of patients such as disease activity index, autoimmune antibodies, and inflammatory marker levels were collected.

**Results:**

A total of 102 SLE patients were included, 24 of which had retinopathy, and 78 had unaffected retina. Wide-field OCTA could effectively detect retinal vascular obstruction, non-perfusion area, and morphological abnormalities in patients with lupus retinopathy. SLE patients without retinopathy had significantly higher retinal superficial vessel density (SVD) in foveal (P=0.02), para-foveal temporal (P=0.01), nasal (P=0.01), peripheral foveal temporal (P=0.02), and inferior areas (P=0.02), as well as subregion temporal (P=0.01) and inferior areas (P=0.03) when compared with healthy controls (n=65 eyes from 65 participants). The area under curve (AUC) value of subregion inferior SVD combined parafoveal temporal SVD was up to 0.70. There was a significantly positive correlation between SVD and disease activity in SLE without retinopathy group. Patients with severe activity had the most significant increase in SVD.

**Conclusion:**

Wide-field OCTA can provide a relatively comprehensive assessment of the retinal vasculature in SLE. In the absence of pathological changes of the retina, the SVD was significantly increased and was positively correlated with the disease activity of SLE.

## Introduction

Systemic lupus erythematosus (SLE) is an autoimmune disease with a prevalence of 20-150 per 100,000 people and can involve multiple organs (1). SLE predominantly affects women of childbearing age with heterogeneous clinical expressions and courses (2). About one-third of SLE patients may suffer from ocular manifestations. All the parts of the eye may be involved, including external, anterior, posterior, and neuro-ophthalmic structures (3).

One of the most common causes of vision loss in SLE is lupus retinopathy (LR), with a prevalence that varies widely between 3% and 29%, which may be due to the deposition of immunoglobulin(Ig) G immune complexes causing vasculitis or antiphospholipid-induced thrombosis (4). LR is well recognized as an accurate reflection of SLE activity and has a significant association with lower survival rates or poor prognosis (5). Multimodal ophthalmic imaging plays a crucial role in detecting the retinopathy. Moreover, with the quantitative function of some imaging techniques, especially optical coherence tomography (OCT) and OCT-angiography (OCTA), changes in the retinal microstructure or vasculature have been detected in SLE patients without LR (6, 7). These changes might be a potential marker for the diagnosis of SLE and have close relationships with systemic organs’ involvement or disease activity. Therefore, comprehensive evaluation of the retina is important for the early detection of retinopathy and abnormalities in the preclinical stage, which can contribute to early treatment, or even prevent irreversible ocular and systemic complications.

However, there are issues that remain to be addressed. First, most of the previous publications had small sample sizes, in which the disease activity and clinical stage of the included subjects were inconsistent with each other, making the conclusions impossible to generalize. Second, several studies used OCTA to quantitatively evaluate the retinal vasculature in patients with SLE, however, they had a small field of view (3x3 mm or 6x6 mm) and the evaluation of peripheral vessels has not been reported in the literature (8). Third, there were case reports that demonstrated an enlarged field to visualize the peripheral retinal lesions by montaging images in SLE patients, which is time-consuming and requires the perfect cooperation between examiner and patients.

In this study, we aimed to evaluate the retinal vascular characteristics of SLE in a relatively large sample size with a novel wide-field OCTA device, which can evaluate the retina in a field of view of 24×20 mm by a single capture (9, 10). We described the retinal vascular characteristics of LR using this equipment. Further, we conducted a quantitative analysis for those yet to undergo pathological changes in the retina and correlated the retinal metrics with systemic indicators, such as disease activity index, the presence of autoantibodies, and other organs’ involvement. We hope that this may not only provide a novel way to evaluate the LR and achieve the early detection of fundus abnormalities but also help make diagnoses and evaluate the systemic condition of SLE.

## Materials and methods

### Study subjects

This prospective observational cross-sectional study was conducted in Peking Union Medical College Hospital from November 2022 to April 2023. It has been approved by the Ethics Committee of Peking Union Medical College Hospital (ethics number: I-22PJ1024). This study followed the principle of the Declaration of Helsinki. All subjects provided informed consent at enrollment.

The inclusion criteria were as follows: 1) age ≥ 18 years old; 2) meeting the classification criteria of the 2019 European College of Rheumatology/American College of Rheumatology (EULAR/ACR) (11); 3) being willing to participate in this study. Meanwhile, age- and gender-matched individuals were selected as controls. The exclusion criteria in both groups were: 1) comorbid with other autoimmune diseases; 2) refractive media opacity or the image scan quality index ≤ 6 due to the poor cooperation of the patients; 3) comorbid with diabetic retinopathy (DR), macular degeneration, glaucoma, retinal detachment, hypertensive retinopathy and other eye diseases; 4) History of ocular trauma or previous treatment with vitrectomy and fundus laser surgery; 5) diagnosis of chloroquine/hydroxychloroquine-related chororetinal lesions; 6) the spherical equivalent (SE) ≥ ± 4.00 diopters (D).

### Data collection

Clinical information and laboratory data within one week of the ophthalmic examination were collected. The contents include 1) demographic data: ethnicity, gender, age, height, and weight; 2) SLE-related information: SLE disease activity index-2000 (SLEDAI-2K), disease duration, involved organs, presence of autoantibodies, complement level, erythrocyte sedimentation rate (ESR), C-reactive protein (CRP), antiphospholipid antibodies (aPLs), lupus coagulants (LA) and treatment; 3) past medical history and medications, including diabetes, hypertension, hyperlipidemia, and coronary heart disease.

SLE patients without retinopathy were then divided into three subgroups according to the score of SLEDAI-2K: mild group (SLEDAI-2K ≤ 6); moderate group (7 < SLEDAI-2K ≤ 12); severe group (SLEDAI-2K ≥ 13). A low complement level was defined as either complement 3 (C3) < 0.73 g/L or complement 4 (C4) <0.1 g/L.

### Ophthalmic examination

All subjects underwent a comprehensive ocular examination bilaterally including best corrected visual acuity (BCVA), non-contact intraocular pressure (IOP) measurement, axial length examination, slit lamp examination before and after dilation of pupils, and OCT. The BCVA was converted to the Logarithm of the Minimum Angle of Resolution (logMAR) visual acuity recordings.

OCTA examination used the newly developed swept-source OCT (SS-OCT) equipment (BM-400K BMizar; TowardPi Medical Technology, Inc., Beijing, China), with an acquisition speed of 400,000 A-scans/second, a laser light wavelength of 1060 nm, a bandwidth of 100 nm as well as optical resolution of axial and lateral tissues of 3.8 µm and 10 µm, respectively. The 24x20mm OCTA scan, which was composed of 1,280 cross-sectional images (B-scans) and centered on the fovea, corresponded to a 120° angular field of view. Besides, the optic disc OCTA (6x6mm) scan, centered on the optic nerve was obtained.

### Evaluation of OCTA images

OCTA images were investigated by two ophthalmologists and all of the inconsistencies were resolved after discussion with the corresponding authors. Vessel occlusion, non-perfusion area, subretinal fluid, retinal hemorrhage, vascular tangling, and tortuosity were recorded. After a comprehensive ophthalmic examination, the patients were divided into two groups: the LR group and the SLE without retinopathy group. LR group was defined as the presence of any of the following lesions in either eye: cotton wool spots, microaneurysms, hard exudates, retinal vascular attenuation, dot hemorrhages, vessel occlusion, non-perfusion area, and retinal vasculitis. The SLE without retinopathy group was defined as none of the retinal abnormalities above was detected in either eye.

For the SLE patients without retinopathy group and control group, data from ophthalmic examination of both eyes were evaluated and the BCVA results from the worst eye were finally analyzed. The superficial layer of the retina was delineated by the internal limiting membrane (ILM) to 9 µm below the inner plexiform layer (IPL). The deep layer was delineated by 6 µm below the IPL to 9 µm below the outer plexiform layer(OPL). The inner retina was defined from ILM to IPL, and the outer retina from the IPL to Bruch’s membrane. These sub-layers were stratified by the built-in software of the device and then the retinal thickness and vessel density (VD) were measured automatically. Specifically, the following parameters were included: 1) fovea avascular zone (FAZ) parameters, including FAZ area, perimeter, acircularity index, and VD of the 300µm range around FAZ(FD-300); 2) The foveal superficial VD (SVD) and deep VD (DVD); the parafovea temporal, superior, nasal and inferior SVD and DVD (the parafovea area was defined as an annular with an outer diameter of 3.0 mm and an inner diameter of 1.0 mm); the perifovea temporal, superior, nasal and inferior SVD and DVD (the peripheral area was defined as an annular area with an outer diameter of 6.0 mm and inner diameter of 3.0 mm); 3) subregion SVD and DVD. The 24x20mm images were divided by the software into 9 subregions: superior nasal (SN), superior (S), superior temporal (ST), nasal (N), central macular area (C), temporal (T), inferior nasal (IN), inferior (I), and inferior temporal (IT) regions, as shown in **Supplementary File 1**; 4) the thickness of the inner and outer layers of the retina, including the early treatment diabetic retinopathy study (ETDRS) area and nine sub-regions; 5) retinal nerve fiber layer (RNFL) thickness and VD in the peripapillary area. The optic disc OCTA scan was divided into eight sub-regions according to the Garway-head partition method (12), and then merged into four areas: the temporal, superior, nasal, and inferior areas. The annulus had an inner diameter of 2.0 mm and an outer diameter of 4.0 mm, respectively (**Supplementary File 2**).

### Statistical analysis

SPSS (version 22.0; SPSS I nc., Chicago, IL, USA) was used for statistical analysis and GraphPad Prism (version 5, CA, USA) was used for making graphs. The continuous variables with normal distribution were documented with the mean (standard deviation, SD), otherwise the median (interquartile range, IQR) was used for those without normal distribution. For the category data, the quantity (percentage) was used to represent. Comparisons of continuous variables were conducted using independent samples t-test, one-way ANOVA test, Mann-Whitney U test, or Kruskal-Wallis test, as appropriate. For categorical data, the chi-square test or Fisher’s exact test was used. Associations between continuous variables were determined by Pearson or Spearman correlation analysis. Receiver operator characteristic (ROC) analysis was used to determine the area under the curve (AUC), sensitivity, specificity, and cutoff values. Multivariate analysis was conducted if there were significant findings in the univariate analysis. A P value <0.05 was considered statistically significant.

## Results

### General characteristics

OCTA images were collected from 102 SLE patients with a median age of 34.5 years old. There were 87 (85.3%) patients who were women. As for disease activity, the mean SLEDAI-2K score was 11.88 ± 7.10. 20 of these patients (19.61%) belonged to the mild group, 38 (37.25%) belonged to the moderate group and 43 (42.16%) belonged to the severe group. An additional 65 healthy individuals were included as the control group. The demographic and clinical characteristics of the patients are shown in **Table 1**.

**Table 1:** Demographic and clinical characteristics of the included patients with SLE.

	SLE(n=102)
Age (years), median (IQR)	34.50(19.25)
Gender (female), n (%)	87(85.30)
BMI, median (IQR)	22.26(4.05)
Duration of SLE (years), median (IQR)	4.5(12.42)
SLEDAI-2000, mean (SD)	11.88(7.10)
Disease activity group (mild/moderate/severe)	20/38/43(19.60/37.30/42.20)
Laboratory indicators	
aPLs or LA, n (%)	39(38.20)
Anti-Smith antibody, n (%)	27(26.50)
Anti-dsDNA antibody, n (%)	58(56.90)
Low complement, n (%)	65(63.70)
C3 (g/L), mean (SD)	0.71(0.29)
C4 (g/L), median (IQR)	0.10(0.10)
ESR (mm/h), median (IQR)	18.00(24.50)
CRP (mg/L),median (IQR)	1.30(6.31)
Involved organs, n (%)	
Skin	12(11.80)
Hematological system	62(60.80)
Neuropsychiatric system	32(31.40)
Kidney	64(62.70)
Polyplasma membrane cavity effusion	16(15.70)
Lung	23(22.50)
Cardiovascular system	21(20.60)
Secondary APS	21(20.60)
Treatment	
Glucocorticoids, n (%)	102(100.00)
HCQ, n(%)	65(63.80)
Azathioprine, n (%)	8(7.80)
Mycophenolate mofetil, n (%)	52(51.00)
Cyclophosphamide, n (%)	56(54.90)
Tacrolimus, n (%)	31(30.40)
Ciclosporin,n(%)	5(4.90)
Rituximab, and n (%)	7(6.90)
Belizumab, n (%)	13(12.70)
IVIG, n(%)	19(18.60)
Ophthalmic conditions	
BCVA (logMAR), median (IQR)	0.00(0.10)
IOP (mmHg), median (IQR)	14.65(3.52)
SE, median (IQR)	0.00(2.00)

APLs, Anti phospholipid antibodies; BCVA, Best corrected visual acuity; BMI, Body mass index; CRP, C-reactive protein; ESR, Erythrocyte sedimentation rate; HCQ, Hydroxychloroquine; IOP, Intraocular pressure; IQR, interquartile range; IVIG, Intravenous immunoglobulin; LA, lupus anticoagulant; SD, Standard deviation; SE, Spherical equivalent; SLE, Systemic lupus erythematosus; SLEDAI, Systemic lupus erythematosus disease activity index.

Among them, 24 patients had retinal involvement, manifesting the sign of cotton wool spots, retinal hemorrhage, capillary non-perfusion area, or vessel occlusions. Compared with patients without retinopathy (n=78), the LR group had significantly higher SLEDAI-2K score (15.38 ± 7.48 vs 10.73 ± 6.72 respectively, P=0.005) and anti-double stranded Deoxyribonucleic acid (anti-ds-DNA) antibody positive proportion (75.00% vs 51.30% respectively, P=0.04), as well as lower BCVA (logMAR 0.30 vs 0.00 respectively, P<0.001). When making comparisons of clinical and demographic features between the SLE without retinopathy group and the control group with healthy eyes (n=65), the previous had significantly higher proportions of hypertension (37.20% vs 15.40% respectively, P=0.003) and hyperlipidemia (28.20% vs 0.00% respectively, P<0.001). There were no significant differences in age, gender, BCVA, IOP, refractive errors, concomitant diabetes, and coronary heart diseases. The demographic and clinical characteristics of the different groups are shown in **Supplementary File 3**.

### Wide-field OCTA features of lupus retinopathy

**Figure 1:**
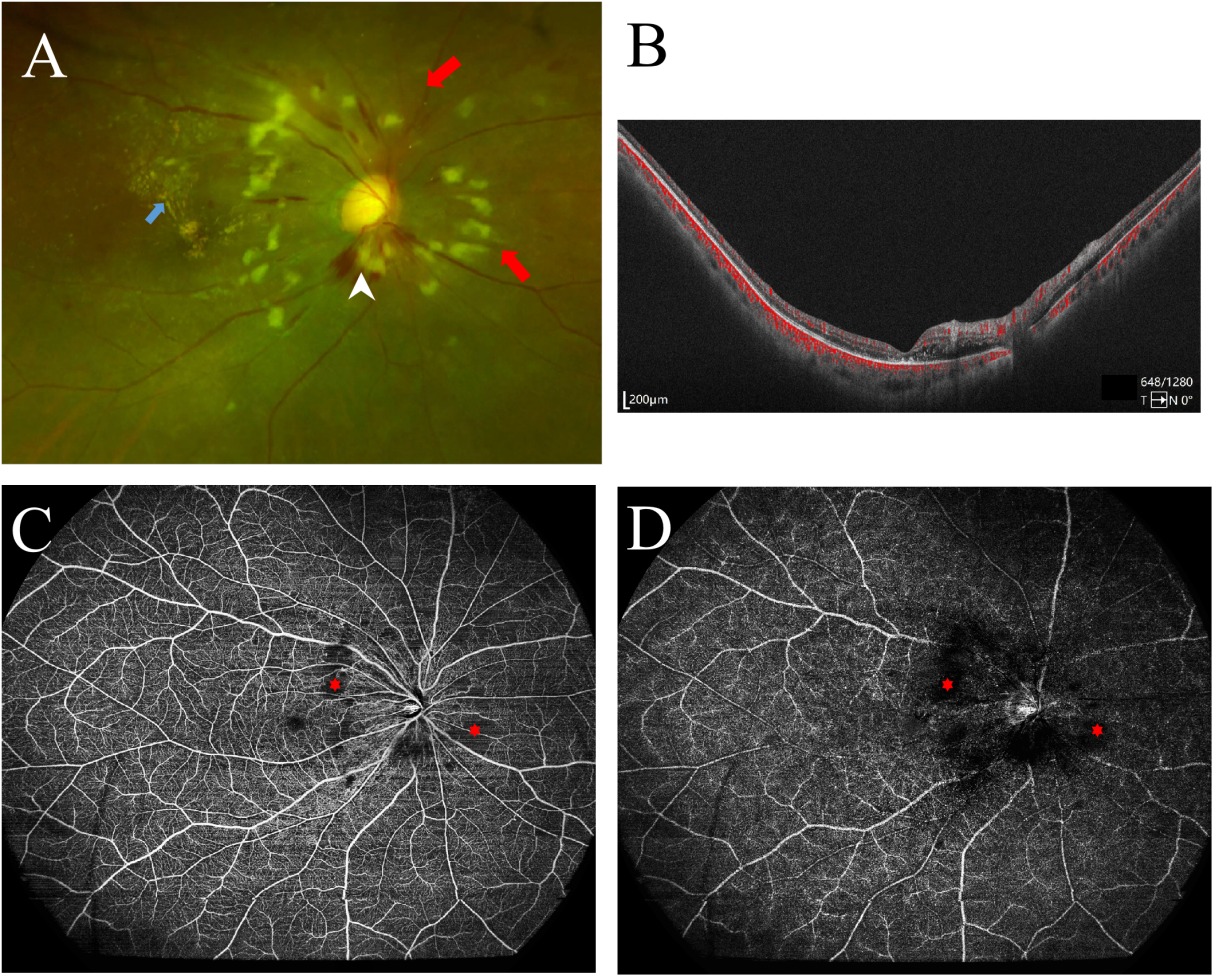
Ocular fundus imaging of systemic lupus erythematosus with retinopathy. **(A)** Ultra-wide-field color fundus photographs showed multiple cotton wool spots (red arrows), exudation (blue arrow), and retinal hemorrhage (white arrowhead); **(B)** Optical coherent tomography angiography (OCTA) B-scan revealed retinal edema and increased thickness. **(C)** OCTA enface image revealed the devoid signal in the superficial retina capillary plexus, indicating non-perfusion areas (asterisks), corresponding to the cotton wool spots on the color fundus photograph. **(D)** The deep retinal capillary network of the right eye showed the devoid signal also (asterisks).

**Figure 2:**
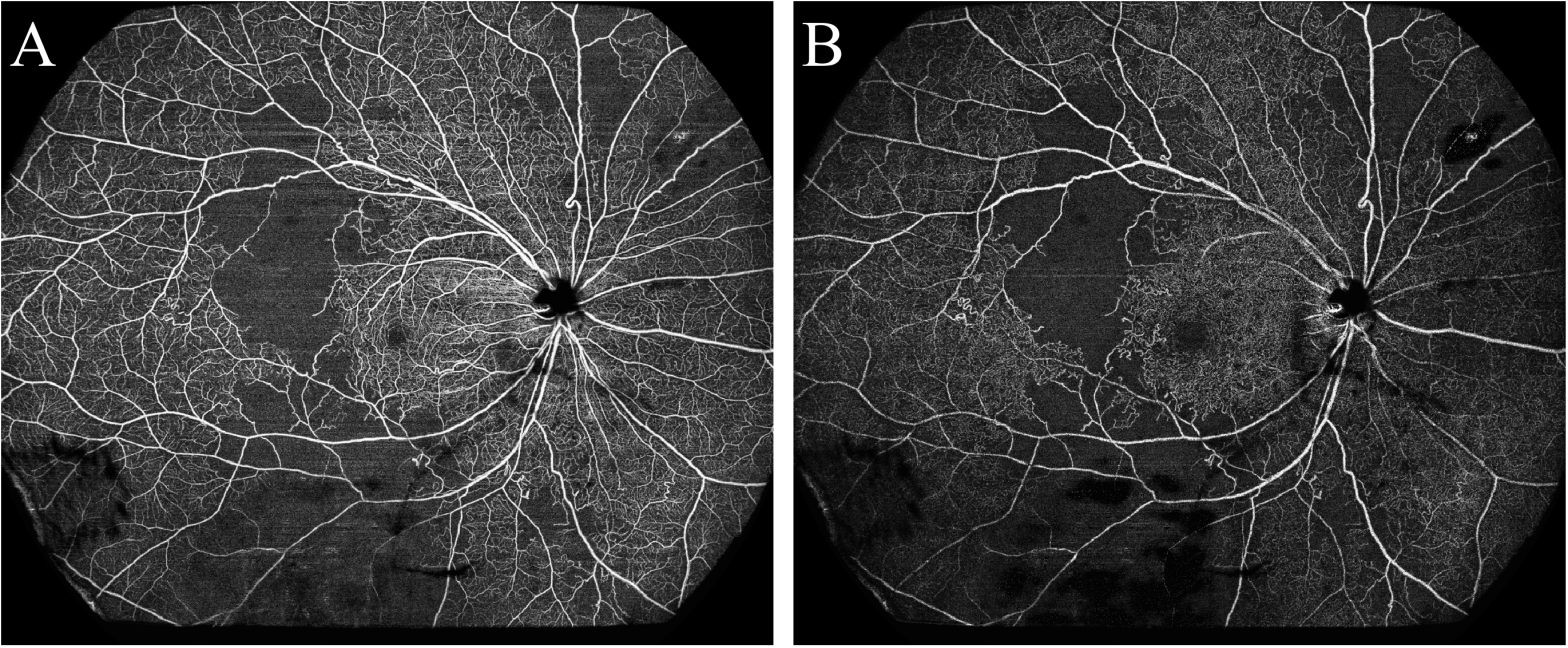
Optical coherence tomography angiography detected a large area of retinal non-perfusion (asterisks) in a patient with systemic lupus erythematosus. **(A)** Superficial retinal vasculature; **(B)** Deep retinal vasculature.

As is shown in **Figures 1** and **2**, the ultra-wide-field color fundus photograph showed multiple cotton wool spots and several retinal hemorrhages around the optic disc, with hard exudates in the posterior pole. OCTA enface images clearly demonstrated the destruction of FAZ, its enlargement area, and morphological changes. Besides, it could exhibit capillary non-perfusion areas and vessel occlusions at the superficial and deep layers. B-scan images showed increased hyper-reflectivity in the inner retinal layers and increased thickness due to retinal edema.

### Retinal vasculature changes in SLE eyes without retinopathy

OCTA images of SLE eyes without retinopathy could not be differentiated from the healthy controls just by observation (**Supplementary File 4**). However, through quantitative analysis, we found several significant differences between the SLE without retinopathy group and the control group, especially in the aspects of retinal vasculature (**Table 2**).

**Table 2:** Differences in retinal microvasculature parameters between SLE without retinopathy group and control group.

	SLE without retinopathy (n=78)	Controls (n=65)	P	P†
FAZ related parameters				
Area, mm2	0.27(0.17)	0.27(0.25)	0.39	/
Perimeter,mm	2.27(0.85)	2.35(1.05)	0.69	/
Acircularity	0.65(0.24)	0.68(0.20)	0.94	/
FD-300, %	15.58(5.85)*	13.51(5.94)*	0.04	0.30
Fovea SVD, %	9.19(4.02)*	7.22(4.40)*	0.006	0.02
Parafovea SVD, %				
Temporal	22.63(4.42)*	19.72(5.10)*	<0.001	0.01
Superior	26.79(5.19)*	25.17(5.07)*	0.06	/
Nasal	22.00(6.00)	19.00(6.00)	0.001	0.01
Inferior	28.00(5.00)	26.00(8.00)	0.01	0.12
Perifovea SVD, %				
Temporal	30.00(7.00)	28.00(6.00)	0.001	0.02
Superior	34.00(6.00)	33.55(4.93)*	0.99	/
Nasal	35.00(6.00)	32.00(6.00)	0.004	0.08
Inferior	37.50(7.00)	33.72(5.03)*	0.001	0.02
Fovea DVD, %	21.00(8.00)	18.00(12.00)	0.44	/
Parafovea DVD, %				
Temporal	29.00(12.00)	29.00(13.00)	0.48	/
Superior	29.00(29.00)	28.00(12.00)	0.18	/
Nasal	29.00(13.00)	29.00(13.00)	0.38	/
Inferior	29.00(12.00)	29.00(14.00)	0.62	/
Perifovea DVD, %				
Temporal	29.00(11.00)	29.00(13.00)	0.37	/
Superior	29.00(12.00)	28.00(12.00)	0.19	/
Nasal	29.00(12.00)	29.00(13.00)	0.42	/
Inferior	29.00(12.00)	28.00(14.00)	0.21	/
Subregion SVD,%				
Whole	24.71(3.24)*	23.76(2.43)*	0.049	0.20
SN	23.00(12.00)	24.54(5.75)*	0.09	/
S	29.32(4.42)*	28.92(3.75)*	0.57	/
ST	22.50(10.00)	21.82(5.68)*	0.91	/
N	29.40(5.91)*	30.00(9.00)	0.90	/
C	34.00(5.00)	32.00(5.00)	0.004	0.07
T	26.00(9.00)	23.31(4.79)*	0.01	0.01
IN	20.00(7.00)	16.78(5.13)*	0.008	0.06
I	24.50(10.00)	23.68(6.09)*	0.002	0.03
IT	18.00(9.00)	17.00(4.29)	0.16	/
Subregion DVD, %				
Whole	27.22(8.06)	26.67(9.00)	0.12	/
SN	27.00(9.00)	27.00(10.00)	0.69	/
S	29.00(8.00)	29.00(11.00)	0.16	/
ST	27.50(8.00)	27.00(9.00)	0.28	/
N	29.00(8.00)	28.00(9.00)	0.05	/
C	29.00(11.00)	29.00(11.00)	0.06	/
T	29.00(8.00)	27.00(9.00)	0.05	/
IN	25.00(8.00)	22.00(8.00)	0.05	/
I	25.00(8.00)	25.00(8.00)	0.05	/
IT	23.00(8.00)	24.00(9.00)	0.43	/

DVD, Deep vessel density; FAZ, Fovea avascular zone; FD-300, Vessel density of the 300µm range around FAZ; SVD, Superficial vessel density; SLE, Systemic lupus erythematosus; VD, Vessel density; SN, Superior nasal; S, Superior; ST, Superior temporal; N, Nasal; C, Central; T, Temporal; IN, Inferior nasal; I, Inferior; IT, Inferior temporal.

*Represented the mean (standard deviation), and the remaining quantitative data were median (interquartile range);

†Adjusted the factors of hyperlipidemia and hypertension.

First, regarding the central macula, we found that SVD in the fovea, parafovea temporal and nasal, perifovea temporal and nasal areas was significantly increased in the SLE without retinopathy group after adjusting the confounding factors of hyperlipidemia and hypertension (P<0.05). However, there was no significant statistical difference in DVD and FD-300 between the two groups after the statistical adjustment. No differences were found in the FAZ area, acircularity, and perimeter.

Second, when dividing the wide-field OCTA images into 9 subregions, we found that after adjusting the confounding factors the SVD was significantly increased in the temporal and inferior regions in the SLE without retinopathy group (P<0.05). No statistical differences were found in the SVD of the whole image and the remaining six subregions. And there were no significant differences in DVD between the two groups.

### Retinal thickness and peripapillary RNFL structural changes in SLE eyes without retinopathy

Compared with the control group, the thickness of both inner and outer retinal layers in the central macula of the SLE group had no significant changes. In terms of 9 subregions, the inner retinal thickness of the subregion IT and subregion ST was significantly higher in the SLE group than in the control group (P<0.05, **Supplementary File 5**).

Additionally, there was no significant difference between the SLE without retinopathy group and the control group in the RNFL VD and thickness in the nasal, superior, temporal, and lower regions after adjusting for the confounding factors of hypertension and hyperlipidemia (**Supplementary File 6**).

### ROC curve analysis

**Figure 3:**
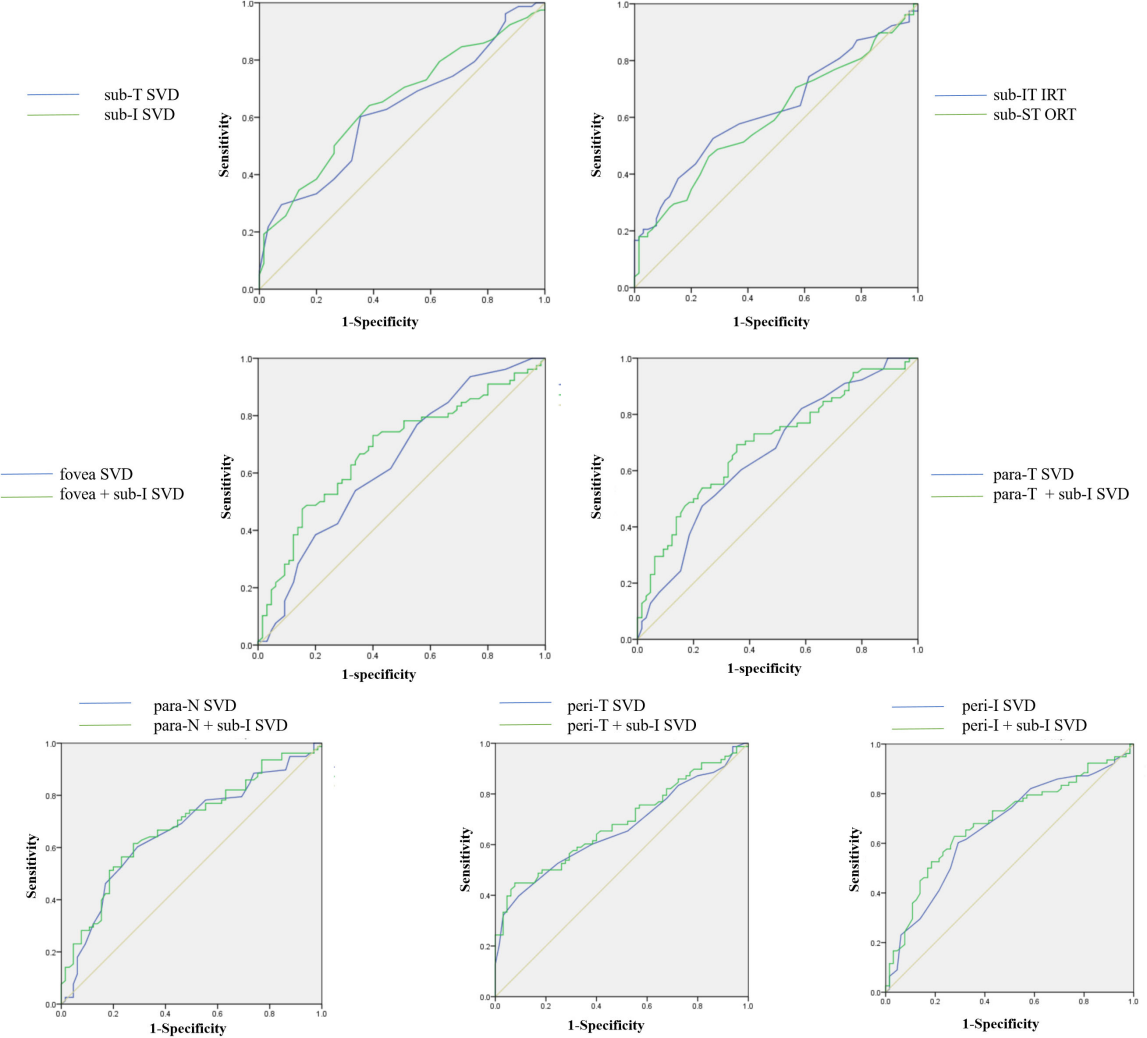
ROC curves of retinal vessel density and thickness in the regions with significant changes. SVD, superficial vessel density; IRT, Inner retinal thickness; ORT, Outer retinal thickness; Sub, Subregion; Para, Parafovea; Peri, Perifovea; T, Temporal; I, Inferior; IT, Inferior temporal; ST, Superior temporal; N, Nasal.

The ROC curve of the statistically significant parameters is presented in **Figure 3**. The AUC values are shown in **Table 3**. Parafovea and perifovea AUC values were all above 0.65 (P=0.001). The AUC of subregion I SVD and subregion T was 0.65 (P=0.002) and 0.62 (P=0.01) respectively. The AUC values of retinal thickness were lower than those of SVD. When combining the fovea SVD with the peripheral SVD, the AUC values were all improved over the single parameters. Among them, subregion I SVD combined with parafovea temporal SVD was the highest (AUC= 0.70, P <0.001).

**Table 3:** ROC curve analysis of retinal vessel density and thickness.

	AUC value	95%CI	P	Cutoff value	Sensitivity	Specificity
Fovea SVD	0.64	0.54-0.73	0.004	6.50	0.77	0.45
Parafovea SVD						
Temporal	0.66	0.57-0.75	0.001	23.50	0.47	0.77
Nasal	0.67	0.58-0.76	0.001	19.50	0.69	0.54
Perifovea SVD						
Temporal	0.66	0.58-0.75	0.001	31.50	0.40	0.91
Nasal	0.66	0.57-0.75	0.001	36.50	0.60	0.71
Subregion SVD						
Temporal	0.62	0.53-0.71	0.01	24.50	0.60	0.65
Inferior	0.65	0.56-0.74	0.00	22.50	0.64	0.62
Subregion I +fovea SVD	0.68	0.59-0.77	<0.001	31.14	0.73	0.60
Subregion I + parafovea SVD						
Temporal	0.70	0.61-0.78	<0.001	59.31	0.69	0.65
Inferior	0.68	0.60-0.77	<0.001	50.60	0.62	0.72
Subregion I +perifovea SVD						
Temporal	0.69	0.60-0.77	<0.001	82.48	0.45	0.92
Inferior	0.69	0.60-0.77	<0.001	67.00	0.63	0.72
Subregion IT IRT	0.63	0.54-0.72	0.01	52.50	0.53	0.72
Subregion ST ORT	0.60	0.51-0.69	0.05	167.50	0.46	0.74

ROC, Receiver operating characteristic; SVD, Superficial vessel density; IRT, Inner retinal thickness; ORT, Outer retinal thickness; I, Inferior; IT, Inferior temporal; ST, Superior temporal.

### Correlation analysis between retinal vasculature features and systemic indicators in SLE eyes without retinopathy

According to the Pearson/Spearman correlation analysis (**Supplementary File 7**), the whole image SVD had a significantly positive association with SLEDAI-2K (r =0.263, P=0.020). Parafovea temporal SVD had a significantly positive association with SLEDAI-2K (r=0.303, P=0.007). The FAZ perimeter showed a significantly positive correlation with the ESR (r=0.276, P=0.015). C3 showed a significantly negative correlation with SVD in the area of SN, N, and IT; C4 showed a significantly negative correlation with SVD of the whole image (r=-0.28, P=0.013) and in the area of SN, N, I, and IT. FD-300, parafoveal DVD, and DVD in subregions IN, I, and IT showed significant negative correlations with the duration of Hydroxychloroquine (HCQ) treatment. There was a significantly negative correlation between age and SVD in the subregion SN; while the duration of SLE and CRP had no significant associations with OCTA vascular parameters in this study.

As for categorical data (**Supplementary File 8**), it was found that SLE patients with lower complements had significantly higher SVD and DVD in the area of SN, N, and IT compared with patients with normal complements; the whole image SVD was also significantly higher in lower complement group (P=0.028). In patients with positive antiphospholipid syndrome (APS)-related antibodies, FD-300, and SVD were increased but without significant differences. In patients with neuropsychiatric SLE (NPSLE), the FAZ acircularity was significantly decreased than those without NPSLE (P=0.004). In patients with cardiac involvement, the SVD was significantly higher in most areas including the whole image than those without cardiac involvement (P=0.02). In addition, we made comparisons on the presence or absence of renal involvement, blood system involvement, and secondary APS, but found no significant differences.

### Multiple linear regression analysis

We conducted a multiple linear regression analysis regarding the seven regions that had significant differences between the SLE without retinopathy group and the control group. Considering the significant results obtained from univariate analysis, independence between variables and possible influencing factors based on clinical practice, SLEDAI-2K score, duration of HCQ treatment, APS-related antibodies, and ESR were included as independent variables to explore the influencing factors of increased VD in the SLE without retinopathy group. As shown in **Table 4**, SVD in the parafovea temporal area, perifoveal temporal area, and area in subregion T had significantly positive association with SLEDAI-2K score (P=0.003, P=0.04, P=0.03 respectively). HCQ showed significant association with subregion T SVD and ESR showed significant correlation with parafovea inferior SVD.

**Table 4:** Multiple linear regression analysis regarding the seven regions which had significant differences between SLE without retinopathy group and control group.

	Variables	β	95%CI	P
Parafovea temporal SVD	SLEDAI	0.23	0.08-0.38	0.003
	HCQ	0.12	-0.11-0.35	0.31
	aPLs/LA (+)	0.47	-1.44-2.38	0.63
	ESR	-0.007	-0.05-0.03	0.73
Perifovea temporal SVD	SLEDAI	0.18	0.01-0.35	0.04
	HCQ	0.19	-0.07-0.45	0.15
	aPLs/LA (+)	0.23	-1.93-2.38	0.84
	ESR	-0.02	-0.06-0.02	0.40
Subregion T SVD	SLEDAI	0.19	0.02-0.36	0.03
	HCQ	0.30	0.04-0.56	0.02
	aPLs/LA (+)	1.05	-1.11-3.20	0.34
	ESR	0.006	-0.04-0.05	0.77
Subregion I SVD	SLEDAI	0.19	-0.02-0.40	0.08
	HCQ	0.07	-0.26-0.39	0.69
	aPLs/LA (+)	2.03	-0.67-4.73	0.14
	ESR	-0.02	-0.07-0.04	0.55
Fovea SVD	SLEDAI	0.11	-0.03-0.25	0.13
	HCQ	-0.05	-0.27-0.16	0.63
	aPLs/LA (+)	0.24	-1.56-2.04	0.80
	ESR	-0.002	-0.04-0.03	0.92
Perifovea inferior SVD	SLEDAI	0.13	-0.06-0.31	0.17
	HCQ	0.08	-0.20-0.35	0.59
	aPLs/LA (+)	0.37	-1.94-2.67	0.76
	ESR	-0.05	-0.09-(-0.001)	0.04
Parafovea nasal SVD	SLEDAI	0.007	-0.17-0.19	0.94
	HCQ	0.11	-0.16-0.38	0.43
	aPLs/LA (+)	0.88	-1.39-3.15	0.45
	ESR	0.01	-0.03-0.06	0.61

aPLs, Antiphospholipid antibodies; ESR, Erythrocyte sedimentation rate; HCQ, Hydroxychloroquine; LA, lupus anticoagulants; SVD, Superficial vessel density; SLE, Systemic lupus erythematosus; SLE, Systemic lupus erythematosus disease activity index.

### Comparison of retinal vasculature features in mild/moderate/severe patients

**Figure 4:**
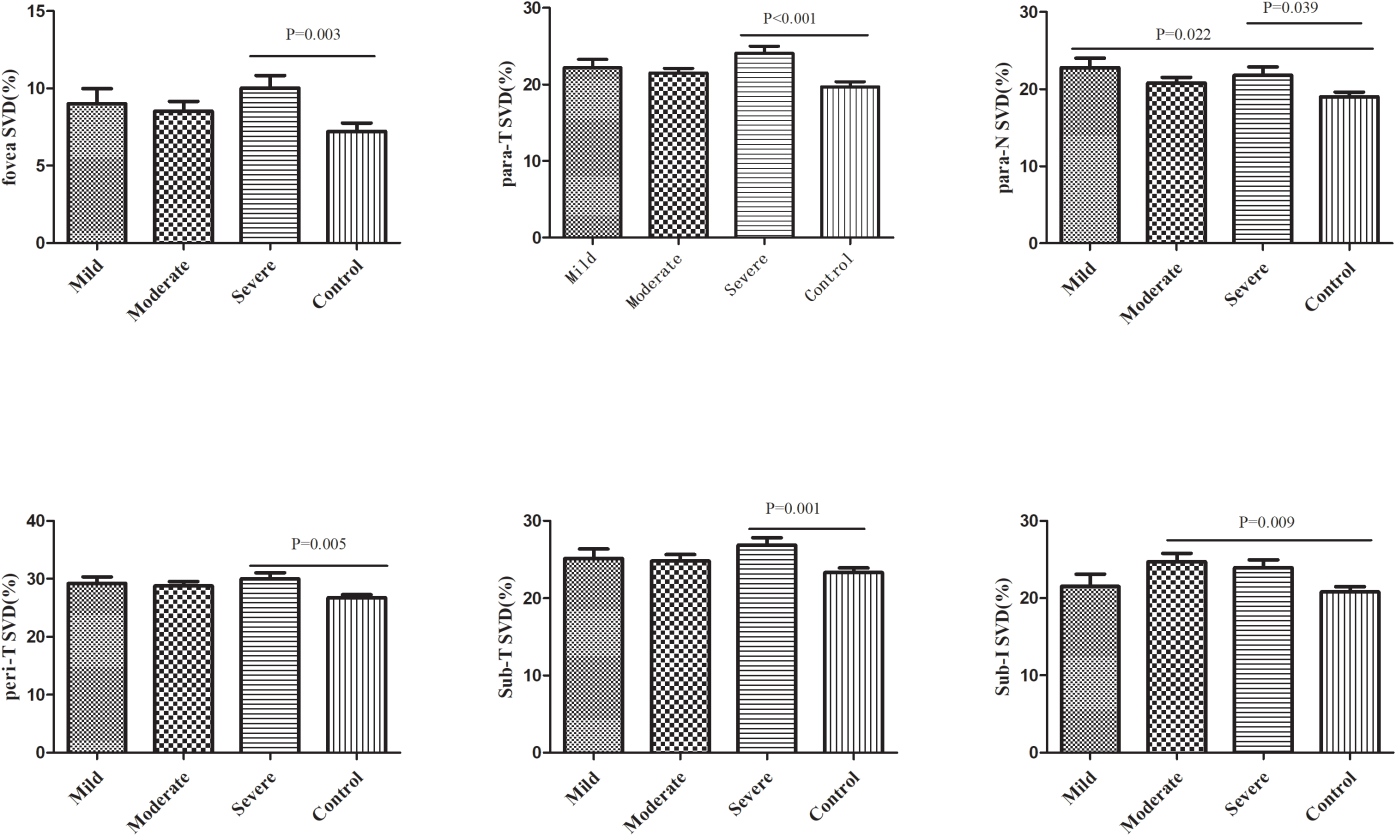
Comparison of retinal vessel density between SLE patients with different disease activity levels and the control group. SVD, superficial vessel density; Para, parafovea; Peri, Perifovea; Sub, Subregion; T, Temporal; N, Nasal.

Subsequently, we divided SLE without retinopathy patients into three groups according to the SLEDAI-2K score: mild, moderate, and severe groups. The clinical characteristics of the three groups are shown in **Supplementary File 9**. A comparison of retinal vasculature among these three groups and controls is shown in **Supplementary File 10**. When conducting pairwise comparison of those 7 regions which had significant differences between SLE without retinopathy group and control group, it was found that SVD of the severe patients in the fovea area (P=0.003), parafovea temporal area (P<0.001), parafovea nasal area (P=0.039), and perifovea temporal area (P=0.005) and subregion T (P=0.001) were significantly higher than the control group. In subregion I, patients with moderate SLE had significantly higher SVD than control group (P=0.009), In the parafovea nasal area, the mild group had significantly higher SVD than the control groups (P=0.022). the remaining pairwise comparisons between groups were not statistically significant (**Figure 4**).

## Discussion

SLE is a systemic autoimmune disease that can involve multiple organs throughout the body, including the retina. Recently, with the development of multimodal ophthalmic imaging techniques, particularly OCT/OCTA, researchers have not only evaluated retinopathy in a more comprehensive way, but also detected the subclinical changes of the retina (13). It has been highlighted that OCT/OCTA could possibly act as a monitoring and prognostic biomarker of kidney injury, cardiovascular disease, cerebral neurodegenerative or vessel disorders in a non-invasive way (14–17). In this study, SLE retinopathy and quantitative parameters of retinal vasculature in patients at subclinical stages were analyzed using a novel type of wide-field OCTA device. The results showed that the wide-field OCTA could effectively evaluate the retinal vasculature and it had a certain diagnostic value in SLE. Besides, it suggested that retinal VD was tightly associated with the disease activity of SLE.

### Assessment of SLE retinopathy using wide-field OCTA

Based on our results, a single capture of 24×20mm OCTA image could clearly reflect retinal vessel perfusion within 120° of the intraocular angle, which was of great importance in discovering the occult non-perfusion area and assessing the severity of retinopathy in patients with SLE. Wide-field OCTA as a non-invasive tool to evaluate the retinal vasculature could reflect more value when encountering patients with severe LN who have contraindications of fluorescein angiography.

### Differences in retinal vessel density between the SLE without retinopathy group and the control group

According to our results, SVD in multiple areas was significantly higher than that in the control group, most of which were concentrated in the central macula. There were no significant differences in DVD and FAZ compared with the control group. These findings were inconsistent with most previous publications, which advocated the decreasing VD in the subclinical retinal stage (8). However, when comparing the clinical characteristics of patients included in different studies, we could find some features that might explain these contradictory results. Firstly, some of the studies did not calculate the subjects’ SLEDAI-2K scores, and their sample sizes were quite small. Thus, the conclusions were not highly generalizable to the entire SLE population (18, 19). Secondly, for articles that evaluated the SLEDAI-2K score or evaluated patients’ disease activity, as shown in **Supplementary File 11**, the SLEDAI-2K reported in the literature was much lower than that in our study (10.73) (13, 20–24). Most SLE patients in the literature were inactive or in mild activity. Therefore, our study which included more severe patients was an important supplement to the existing literature. Thirdly, various OCTA devices in these studies might have subtle impacts on the results.

In this study, we utilized the advantage of a wide-field device to evaluate the peripheral VD and thickness of the retina. It was found that SVD of subregion T and I in SLE without retinopathy group were significantly higher than the control group. IRT ST and ORT IT were also significantly increased in the SLE without retinopathy group. According to the results of ROC curve analysis, we found that the AUC values were improved when combining the VD near the fovea and the peripheral area, with SVD in subregion I combined parafovea temporal area being the highest (AUC=0.70, P<0.001). These suggested that retinal microvascular changes really have a certain significance for the diagnosis of SLE, coinciding with the previous studies. Furthermore, our study proved the value of peripheral retinal changes, which could improve the diagnostic capability when being integrated into the models.

The clinical presentation of SLE is complex and diverse and lacks specificity. Clinicians make a diagnosis of SLE according to the classification criteria at present. The most recent one is described by the 2019 EULAR/ACR (11). However, the diagnosis of SLE is still a serious problem due to its complexity and lack of sufficient understanding of the disease. Many researchers have been dedicated to finding new biomarkers to diagnose SLE (25). Our research might provide insight into future research directions from the ocular fundus perspective.

In addition, we analyzed the peripapillary RNFL thickness and VD and found no significant difference between the SLE without retinopathy group and the control group, which was consistent with the conclusions of Subasi et al.’s study (23).

### Relationship between retinal vasculature changes and SLE disease activity

In this study, correlation analysis was conducted between retinal vasculature parameters in the SLE group and their clinical characteristics. Increased disease activity and inflammation process, reflected by SLEDAI-2K, ESR, and lower complements, seemed to have effects on vasculature. Furthermore, multivariate linear regression analysis was conducted between the 7 regions with significantly increased retinal vascular density in the SLE group and their clinical characteristics. It was found that there was a significant positive correlation between SLEDAI-2K and the SVD of parafoveal temporal area, perifoveal temporal area, and subregion T.

The SLE group was then divided into three groups of mild, moderate, and severe activity based on the SLEDAI-2K score. Among the 7 regions with significantly increased SVD in the SLE group, five regions, including the foveal area (P=0.003), the parafovea temporal area (P<0.001), the parafovea nasal area (P=0.039), the perifovea temporal area (P=0.005), and subregion T (P=0.001), were significantly higher in severe patients than in the control group. The mild group and moderate group only had one region which demonstrated significant differences compared with the control group. These results suggested that the retinal vascular density is increased in SLE patients with severe conditions. Ermurat et al. reported a negative correlation between retinal VD and SLEDAI-2K score in 47 SLE patients (24). However, as discussed above, more than half of the patients included in their study had scores less than 6, while 77% of our subjects had SLEDAI-2K scores greater than 6. Therefore, the heterogeneity of disease activity among the included patients in the two studies might be the main reason for the difference in conclusions.

We speculated several possible explanations for the positive correlation between retinal vascular density and disease activity in SLE patients without retinopathy. First, this might be related to the theory of automatic regulation of retinal microcirculation. The retina relies on local autoregulation to maintain a stable metabolic environment. The blood flow in the retina is often determined by metabolic needs, especially the demand for oxygen (26). In systemic hypoxia conditions, retinal vasodilatation will occur and lead to an increase in retinal blood flow (27). While hyperoxia leads to retinal vasoconstrictor response and results in decreasing blood flow (28). Second, patients with high disease activity are in a state of intense inflammatory response. Hypoxia is a common feature of most inflammation, and conversely, it is also an important factor for inflammation induction or exacerbation (29). Third, there have been clinical studies investigating the vasculature changes by OCTA. Hommer et al. reported that systemic hypoxia could lead to increased VD limited to the superficial vessel layer, while no changes were found in the deep vessel layer. On the contrary, systemic hyperoxia could induce a significant decrease in VD in the deep layer (30). Sousa et al. found that baseline VD increased in hypoxic conditions and subsequently decreased in posthypoxia condition (31). Besides, most of the patients included in this study are in the stage of high-dose steroid therapy, so the effect of water and sodium retention brought about by steroid therapy may also cause the dilation of small retinal blood vessels, thus increasing the blood VD (32). All these factors might contribute to the increase of VD in SLE. Based on our results and literature reports, we speculated that the change of VD in SLE patients without retinopathy may increase at the initial stage and then gradually decrease along the disease course. In patients with SLE retinopathy, it is possible that this process may change very quickly and progress into the microvasculopathy or vaso-occlusive stages in a short time. However, the specific mechanism behind this requires future studies with a larger sample size, as well as evidence from experimental studies.

### Others

ESR was associated with the morphological change of FAZ, exhibiting a significantly positive correlation with FAZ perimeter. Besides, the area increased and the acircularity decreased but without statistical significance. The decrease of C3 and C4 was significantly correlated with the increase in peripheral SVD. As is known, the decrease in complement was a manifestation of SLE disease activity and is an important item of the SLEDAI-2K score system, so the relationship between complement and VD tended to be similar to that between disease activity and VD. The effects of HCQ treatment on retinal vascular density are a controversial topic in the literature. Our results showed that DVD demonstrated a negative correlation with HCQ treatment, which was similar to Forte et al.’s study (19). While some researchers observed an increase of VD in patients who underwent HCQ treatment or no significant differences (33, 34).

Regarding the retinal vasculature changes in the condition of different organs’ involvement, the results showed that SLE patients with cardiac involvement had significantly higher retinal vascular density in some regions than those without cardiac involvement, and patients with NPSLE were lower than those without NPSLE. No significant differences were found between patients with and without involvement of the renal or blood systems. We speculated that patients with cardiac involvement may experience an increase in VD in retinal tissue due to the negative feedback mechanisms of the body, resulting in vascular dilation to compensate for the body’s needs after impaired pumping function of the heart (35). Meanwhile, due to the similarity in anatomy and physiology between the brain and eye, when the central nervous system is involved, retinal tissue may also be about to enter the pathological stage, with FAZ morphology becoming more irregular and VD showing a downward trend. The negative results of the renal system in this study were opposite to Conigliaro et al.’s study (36). Therefore, future studies with larger sample sizes are needed to validate the associations that were found in this study.

### Limitations and future perspectives

This study has the following limitations. Firstly, all the included patients were hospitalized and are not representative enough for the whole group of SLE patients. Secondly, the cross-sectional design precludes inferring the causality, and long-term follow-up of the retinal vasculature changes will help to explore the pathogenesis behind it. Thirdly, though the sample size was the largest when compared with the previous publications, multicenter studies with more subjects are needed in the future to confirm the results.

Recently, several authors have found some biomarkers that might be an alternative or supplementary to the existing tool for assessing SLE disease activity, such as circulating immune complexes (CIC), cytokine levels, and monocyte phenotypes (37, 38). Considering the emerging role of these biomarkers in SLE pathogenesis, it deserves further research in exploring the correlation between retinal changes and these biomarkers from the blood samples. Additionally, it has been reported that there were correlations between nailfold capillaroscopy and VD of OCTA in several immune disorders, such as systemic sclerosis, psoriatic arthritis, and ANCA-vasculitides (39–41). Encouragingly, the combined examination results of ocular microvascular alterations and fingertip perfusion may complement traditional diagnostic methods of these disorders (39). Therefore, it is worth investigating the associations between retinal changes using wide-field OCTA and alterations of the periungual microcirculation using capillaroscopy in patients with SLE in the future.

## Conclusions

In conclusion, this study used wide-field OCTA equipment to observe the retinal vasculature of SLE patients. For SLE patients who had already developed retinopathy, wide-field OCTA imaging was a good evaluation and monitoring method for the severity. For SLE patients whose retina had not yet undergone pathological changes, we found the SVD was significantly increased compared to the control group. These vascular changes might have certain diagnostic efficacy in distinguishing SLE patients from the normal control group. The changes in retinal vasculature in SLE patients were closely related to their systemic conditions. The retinal SVD was significantly increased in individuals with high disease activity. In the future, it is worth further exploring the changes in retinal vasculature in SLE patients during the clinical course, which is of great significance for further understanding its pathogenesis, and making early diagnosis and treatment.

## Data availability statement

The raw data supporting the conclusions of this article will be made available by the authors, without undue reservation.

## Ethics statement

The studies involving humans were approved by the Ethics Committee of Peking Union Medical College Hospital (Ethics number: I-22PJ1024). The studies were conducted in accordance with the local legislation and institutional requirements. The participants provided their written informed consent to participate in this study.

## Author contributions

LM: Conceptualization, Writing – review & editing, Data curation, Formal Analysis, Investigation, Methodology, Project administration, Writing – original draft. LC: Investigation, Methodology, Writing – review & editing. CZ: Conceptualization, Investigation, Writing – review & editing. HC: Conceptualization, Methodology, Writing – review & editing. JY: Investigation, Methodology, Writing – review & editing. YW: Investigation, Methodology, Writing – review & editing. WZ: Data curation, Formal Analysis, Investigation, Writing – review & editing. SC: Software, Writing – review & editing. XZ: Investigation, Methodology, Writing – review & editing. QZ: Investigation, Methodology, Writing – original draft. YC: Conceptualization, Funding acquisition, Supervision, Writing – review & editing, Investigation, Project administration.
