# Supplementary material for: Quantitative assessment of retinal vasculature changes in systemic lupus erythematosus using wide-field OCTA and the correlation with disease activity
Source: Front Immunol. 2024 Jan 29;15:1340224. doi: 10.3389/fimmu.2024.1340224 (PMC10859513; doi:10.3389/fimmu.2024.1340224)
Supplement: Supplementary file 1 [file DataSheet_1.docx]

**Supplementary file 1.** Automatic segmentation and vascular density measurement of ETDRS region and 9 subregions. A&C. Superficial layer of retinal vasculature; B&D. deep layer of retinal vasculature.


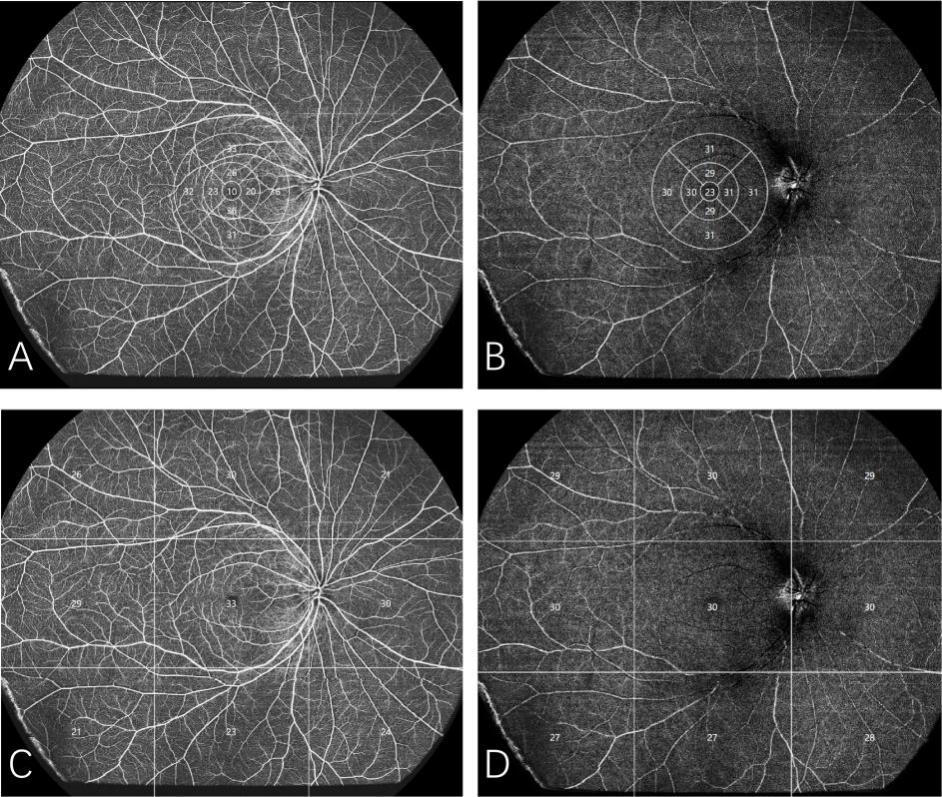


**Supplementary file 2.** Automatic segmentation of retinal nerve fiber layer (RNFL) around the optic disc and the measurement of its thickness and vessel density. A. The vasculature of RNFL; B. The vasculature of the whole retina; C. Schematic diagram of thickness measurement of RNFL; D. Vessel density measurement of RNFL.


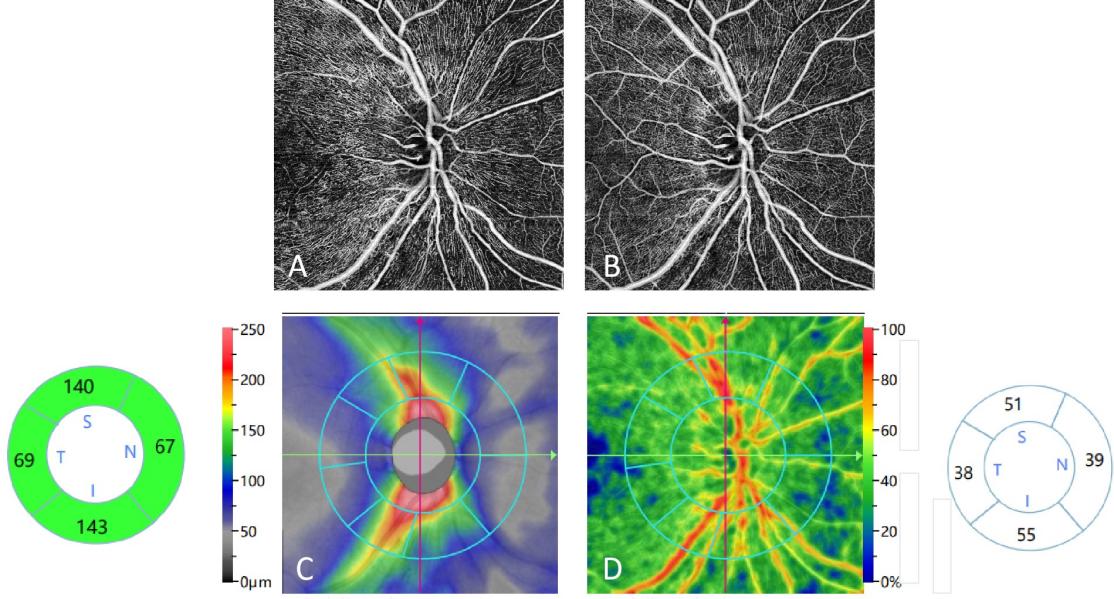


**Supplement file 3.** Comparison of the clinical characteristics of SLE with/without retinal involvement and healthy controls.

|  | SLE retinopathy (n=24) | SLE without retinopathy (n=78) | Control (n=65) | P1 | P2 |
| --- | --- | --- | --- | --- | --- |
| Age (year) | 32.92(12.64)* | 35.00(19.25) | 34.00(14.50) | 0.15 | 0.95 |
| Gender (female), n(%) | 22(91.70) | 65(83.30) | 52(80.00) | 0.51 | 0.61 |
| SLEDAI-2000 | 15.38（7.48）* | 10.73(6.72)* | / | **0.005** | / |
| Disease Activity Group (mild/ moderate/severe,%) | 3/7/14 (12.50/29.20/58.30) | 18/31/29 (23.10/39.70/37.20) | / | 0.17 | / |
| APLs or LA, n (%) | 10(41.70) | 29(37.20) | / | 0.69 | / |
| Anti-Smith antibody, n (%) | 9(37.50) | 18(23.10) | / | 0.17 | / |
| Anti-dsDNA antibody, n (%) | 18(75.00) | 40(51.30) | / | **0.04** | / |
| Low complement, n (%) | 18(75.00) | 47(60.30) | / | 0.18 | / |
| **Comorbidity, n (%)** |  |  |  |  |  |
| Hypertension | 12(50.00) | 29(37.20) | 10(15.40) | 0.27 | **0.003** |
| Hyperlipidemia | 3(12.50) | 22(28.20) | 0(0.00) | 0.10 | **<0.001** |
| Diabetes mellitus | 2(8.30) | 8(10.30) | 6(9.20) | 1.00 | 0.84 |
| Coronary heart disease | 0(0.00) | 4(5.10) | 1(1.50) | 0.58 | 0.23 |
| **Ophthalmic conditions** |  |  |  |  |  |
| BCVA(logMAR) | 0.30(0.82) | 0.00(0.10) | 0.00(0.00) | **<0.001** | 0.14 |
| IOP（mmHg） | 15.30(2.95)* | 14.30(3.55)* | 14.00(2.80) | 0.34 | 0.67 |
| SE | 0(2.66) | 0.00(2.03) | 0.00(2.54) | 0.69 | 0.27 |

APLs: Anti-phospholipid antibodies; BCVA: Best corrected visual acuity; CRP: C-reactive protein; ESR: Erythrocyte sedimentation rate; HCQ: Hydroxychloroquine; IOP: Intraocular pressure; IVIG: Intravenous immunoglobulin; LA: lupus anticoagulant; SE: Spherical equivalent; SLE: Systemic lupus erythematosus; SLEDAI: Systemic lupus erythematosus disease activity index;

P1: SLE retinopathy *vs* without retinopathy group; P2: SLE without retinopathy vs control group;

*Represented the mean (standard deviation), and the remaining quantitative data were median (interquartile range);

**Supplementary file 4.** Wide-field OCTA images of the patients with SLE but without retinopathy.


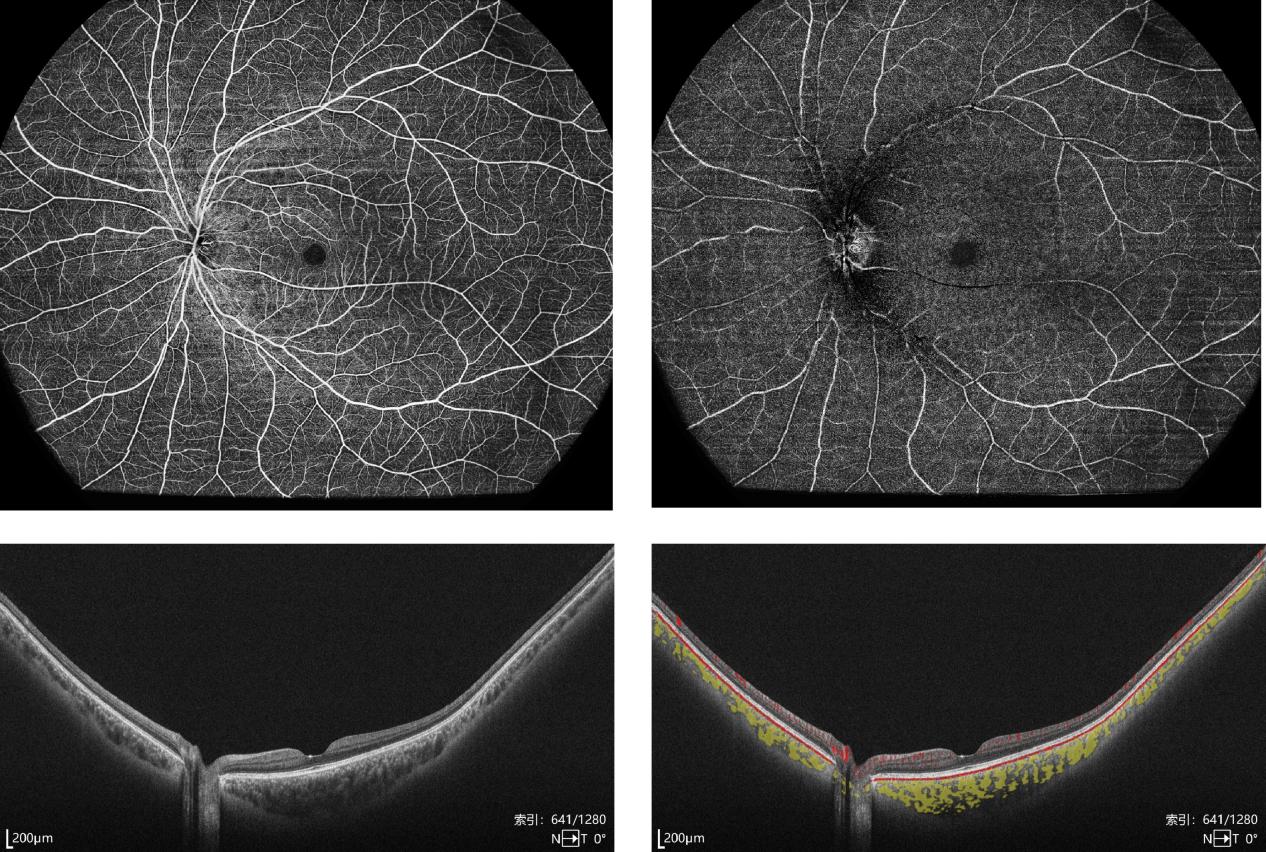


**Supplementary file 5.** Differences in retinal thickness between the SLE without retinopathy group and the control group.

|  | SLE without retinopathy (n=78) | Controls (n=65) | P | P† |
| --- | --- | --- | --- | --- |
| Foveal IRT, μm | 58.50(12.00) | 60.68(11.50)* | 0.57 | / |
| Parafovea IRT, μm |  |  |  |  |
| Temporal | 118.00(9.00) | 120.94(8.24)* | 0.01 | 0.78 |
| Superior | 130.00(11.00) | 133.35(9.31)* | 0.04 | 0.85 |
| Nasal | 127.50(13.00) | 130.89(10.35)* | 0.02 | 0.96 |
| Inferior | 129.00(12.00) | 132.28(10.06)* | 0.15 | / |
| Perifovea IRT, μm |  |  |  |  |
| Temporal | 97.00(10.00) | 98.62(7.50)* | 0.34 | / |
| Superior | 116.50(12.00) | 117.26(9.61)* | 0.35 | / |
| Nasal | 133.00(12.00) | 135.37(11.63)* | 0.22 | / |
| Inferior | 111.00(13.00) | 112.54(10.17)* | 0.38 | / |
| Foveal ORT, μm | 220.00(17.00) | 224.80(10.50)* | 0.06 | / |
| Parafovea ORT, μm |  |  |  |  |
| Temporal | 225.00(11.00) | 225.38(9.21)* | 0.48 | / |
| Superior | 229.85(26.27) | 227.20(9.96)* | 0.61 | / |
| Nasal | 229.00(17.00) | 232.83(12.93)* | 0.23 | / |
| Inferior | 218.50(14.00) | 221.11(9.79)* | 0.35 | / |
| Perifovea ORT, μm |  |  |  |  |
| Temporal | 202.00(13.00) | 202.65(8.79)* | 0.94 | / |
| Superior | 203.83(10.11)* | 204.06(9.58)* | 0.89 | / |
| Nasal | 208.00(17.00) | 206.00(19.00) | 0.78 | / |
| Inferior | 190.23(9.36)* | 189.00(13.00) | 0.76 | / |
| Subregion IRT, μm |  |  |  |  |
| Whole | 72.33(8.31) | 71.51(4.95)* | 0.29 | / |
| SN | 69.00(10.00) | 67.43(7.32)* | 0.44 | / |
| S | 76.00(11.00) | 74.75(6.15)* | 0.33 | / |
| ST | 54.00(9.00) | 53.00(7.00) | 0.20 | / |
| N | 98.00(22.00) | 96.00(14.00) | 0.34 | / |
| C | 114.00(15.00) | 118.63(9.66)* | 0.01 | 0.96 |
| T | 57.00(8.00) | 57.00(5.00) | 0.21 | / |
| IN | 59.50(12.00) | 59.00(7.00) | 0.17 | / |
| I | 68.50(14.00) | 67.43(5.67)* | 0.06 | / |
| IT | 53.00(9.00) | 51.00(5.00) | 0.008 | **0.017** |
| Subregion ORT, μm |  |  |  |  |
| Whole | 174.33(10.11) | 171.89(8.72) | 0.43 | / |
| SN | 170.50(18.00) | 168.00(11.00) | 0.13 | / |
| S | 177.00(14.00) | 175.89(7.85)* | 0.43 | / |
| ST | 166.00(13.00) | 163.00(9.00) | 0.04 | **0.03** |
| N | 175.00(16.00) | 174.00(11.00) | 0.59 | / |
| C | 197.00(16.00) | 195.00(10.00) | 0.40 | / |
| T | 167.00(13.00) | 166.00(9.00) | 0.30 | / |
| IN | 168.00(14.00) | 167.00(11.00) | 0.62 | / |
| I | 172.50(14.00) | 174.55(8.78)* | 0.98 | / |
| IT | 167.00(16.00) | 167.00(11.00) | 0.82 | / |

IRT: Inner retinal thickness; ORT: Outer retinal thickness; SLE: Systemic lupus erythematosus; SN: Superior nasal; S: Superior; ST: Superior temporal; N: Nasal; C: Central; T: Temporal; IN: Inferior nasal; I: Inferior; IT: Inferior temporal.

*Represented the mean (standard deviation), and the remaining quantitative data were median (interquartile range);

†Adjusted the factors of hyperlipidemia and hypertension.

**Supplementary file 6.** Differences in thickness and vessel density of RNFL layer between SLE patients without retinopathy and the control group.

|  | SLE without retinopathy (n=78) | Controls (n=65) | p | p† |
| --- | --- | --- | --- | --- |
| RNFL-thickness,μm |  |  |  |  |
| Whole | 104.37(10.06)* | 105.50(10.88) | 0.227 | / |
| Temporal | 77.00(16.00) | 81.00(14.50) | **0.03** | 0.19 |
| Superior | 126.35(16.25)* | 130.00(18.00) | 0.35 | / |
| Nasal | 80.18(13.58)* | 79.08(12.62)* | 0.62 | / |
| Inferior | 132.18(18.33)* | 134.55(18.46)* | 0.44 | / |
| RNFL-VD, % |  |  |  |  |
| Whole | 45.25(2.56) | 44.43(2.43)* | 0.381 | / |
| Temporal | 40.00(5.00) | 40.00(6.00) | 0.92 | / |
| Superior | 49.50(3.00) | 49.00(4.00) | 0.13 | / |
| Nasal | 42.00(6.00) | 43.00(6.50) | 0.70 | / |
| Inferior | 50.00(3.00) | 49.00(3.00) | 0.37 | / |

RNFL: Retinal nerve fiber layer; VD: Vessel density.

*Represented the mean (standard deviation), and the remaining quantitative data were median (interquartile range);

†Adjusted the factors of hyperlipidemia and hypertension.

Supplementary file 7. Correlation analysis between vessel density and clinical quantitative features in SLE patients without retinopathy.

|  | SLEDAI-2K | | Duration HCQ treatment | | Duration of SLE | | Age | | ESR | | CRP | | C3 | | C4 | |
| --- | --- | --- | --- | --- | --- | --- | --- | --- | --- | --- | --- | --- | --- | --- | --- | --- |
|  | r | P | r | P | r | P | r | P | r | P | r | P | r | P | r | P |
| **FAZ related parameters** |  |  |  |  |  |  |  |  |  |  |  |  |  |  |  |  |
| Area, mm2 | -0.02 | 0.84 | -0.05 | 0.66 | 0.06 | 0.62 | 0.06 | 0.59 | 0.21 | 0.07 | -0.31 | 0.79 | 0.13 | 0.27 | 0.04 | 0.75 |
| Perimeter, mm | 0.04 | 0.71 | -0.15 | 0.20 | 0.003 | 0.98 | 0.03 | 0.82 | 0.28 | **0.02** | -0.02 | 0.89 | 0.03 | 0.80 | 0.04 | 0.76 |
| Acircularity | -0.01 | 0.95 | 0.13 | 0.27 | 0.03 | 0.77 | 0.02 | 0.86 | -0.11 | 0.36 | 0.05 | 0.65 | 0.04 | 0.75 | -0.04 | 0.73 |
| FD-300, % | 0.21 | 0.07* | -0.25 | **0.03** | -0.08 | 0.50 | -0.02 | 0.87 | 0.22 | 0.05 | 0.10 | 0.40 | -0.03 | 0.82* | -0.01 | 0.95* |
| **Fovea SVD, %** | 0.19 | 0.09* | -0.17 | 0.14 | -0.13 | 0.26 | 0.01 | 0.91 | 0.08 | 0.47 | 0.19 | 0.10 | -0.04 | 0.75* | -0.03 | 0.77* |
| **Parafovea SVD, %** |  |  |  |  |  |  |  |  |  |  |  |  |  |  |  |  |
| Temporal | 0.30 | **0.01*** | -0.08 | 0.51 | -0.15 | 0.20 | 0.11 | 0.35 | 0.08 | 0.50 | 0.16 | 0.16 | -0.05 | 0.68* | -0.09 | 0.45* |
| Superior | 0.16 | 0.17* | 0.07 | 0.55 | -0.03 | 0.80 | -0.02 | 0.87 | 0.11 | 0.33 | 0.12 | 0.29 | -0.02 | 0.86* | 0.10 | 0.37* |
| Nasal | 0.01 | 0.96 | -0.08 | 0.48 | 0.09 | 0.45 | 0.08 | 0.51 | -0.02 | 0.87 | 0.09 | 0.45 | 0.06 | 0.63 | -0.09 | 0.43 |
| Inferior | 0.14 | 0.23 | -0.09 | 0.42 | -0.08 | 0.49 | 0.04 | 0.76 | 0.02 | 0.89 | 0.14 | 0.22 | -0.05 | 0.65 | -0.11 | 0.32 |
| **Perifovea SVD, %** |  |  |  |  |  |  |  |  |  |  |  |  |  |  |  |  |
| Temporal | 0.19 | 0.10 | 0.04 | 0.74 | 0.05 | 0.66 | -0.03 | 0.79 | -0.01 | 0.96 | 0.06 | 0.63 | -0.03 | 0.82 | -0.06 | 0.61 |
| Superior | 0.16 | 0.15 | -0.01 | 0.92 | 0.03 | 0.79 | -0.13 | 0.26 | 0.01 | 0.50 | 0.05 | 0.69 | -0.12 | 0.30 | -0.10 | 0.40 |
| Nasal | 0.02 | 0.87 | -0.04 | 0.72 | -0.01 | 0.97 | 0.01 | 0.94 | -0.10 | 0.40 | 0.16 | 0.16 | -0.03 | 0.79 | -0.10 | 0.38 |
| Inferior | 0.04 | 0.74 | <0.01 | 0.97 | 0.04 | 0.72 | -0.06 | 0.63 | -0.08 | 0.49 | -0.02 | 0.88 | -0.03 | 0.80 | -0.16 | 0.15 |
| **Fovea DVD, %** | -0.20 | 0.08 | -0.06 | 0.59 | -0.04 | 0.73 | 0.12 | 0.28 | -0.18 | 0.11 | -0.04 | 0.75 | 0.16 | 0.15 | 0.04 | 0.73 |
| **Parafovea DVD, %** |  |  |  |  |  |  |  |  |  |  |  |  |  |  |  |  |
| Temporal | -0.20 | 0.07 | 0.01 | 0.95 | 0.09 | 0.46 | 0.19 | 0.09 | -0.15 | 0.19 | 0.18 | 0.11 | 0.19 | 0.11 | 0.06 | 0.59 |
| Superior | -0.07 | 0.57 | -0.14 | 0.21 | -0.07 | 0.54 | 0.14 | 0.22 | -0.08 | 0.47 | 0.01 | 0.94 | 0.16 | 0.17 | 0.14 | 0.21 |
| Nasal | -0.10 | 0.37 | -0.01 | 0.95 | -0.10 | 0.38 | 0.03 | 0.80 | -0.15 | 0.20 | 0.002 | 0.98 | 0.20 | 0.09 | 0.03 | 0.79 |
| Inferior | 0.02 | 0.89 | -0.23 | **0.04** | -0.06 | 0.61 | 0.06 | 0.61 | -0.07 | 0.56 | 0.11 | 0.35 | 0.13 | 0.24 | 0.02 | 0.85 |
| **Perifovea DVD, %** |  |  |  |  |  |  |  |  |  |  |  |  |  |  |  |  |
| Temporal | -0.11 | 0.32 | -0.07 | 0.54 | -0.02 | 0.90 | 0.01 | 0.96 | -0.13 | 0.28 | 0.005 | 0.97 | 0.22 | 0.05 | 0.05 | 0.67 |
| Superior | -0.09 | 0.43 | -0.05 | 0.65 | -0.08 | 0.47 | 0.04 | 0.73 | -0.13 | 0.26 | 0.01 | 0.92 | 0.20 | 0.08 | 0.10 | 0.40 |
| Nasal | -0.17 | 0.13 | -0.06 | 0.61 | -0.04 | 0.72 | 0.15 | 0.18 | -0.12 | 0.29 | 0.07 | 0.54 | 0.24 | **0.03** | 0.13 | 0.27 |
| Inferior | -0.03 | 0.77 | -0.11 | 0.35 | -0.15 | 0.20 | 0.03 | 0.79 | -0.01 | 0.94 | 0.17 | 0.13 | 0.22 | 0.057 | 0.12 | 0.30 |
| **Subregion SVD,%** |  |  |  |  |  |  |  |  |  |  |  |  |  |  |  |  |
| Whole | 0.26 | **0.02*** | -0.12 | 0.29 | -0.07 | 0.56 | -0.06 | 0.603 | 0.11 | 0.33 | 0.11 | 0.35 | -0.21 | 0.07* | -0.28 | **0.01*** |
| SN | 0.12 | 0.31 | -0.15 | 0.20 | 0.03 | 0.78 | 0.02 | 0.89 | 0.04 | 0.73 | 0.16 | 0.15 | -0.26 | **0.02** | -0.24 | **0.03** |
| S | 0.15 | 0.20* | -0.13 | 0.27 | -0.11 | 0.33 | -0.26 | **0.02** | 0.04 | 0.70 | 0.03 | 0.80 | -0.05 | 0.70 | -0.04 | 0.71 |
| ST | 0.14 | 0.22 | 0.01 | 0.95 | -0.04 | 0.73 | -0.20 | 0.08 | 0.17 | 0.15 | 0.02 | 0.89 | 0.09 | 0.44 | 0.05 | 0.64 |
| N | 0.08 | 0.49* | -0.15 | 0.20 | 0.06 | 0.59 | 0.18 | 0.11 | 0.13 | 0.24 | 0.15 | 0.18 | -0.23 | **0.04** | -0.26 | **0.02** |
| C | 0.13 | 0.27 | -0.01 | 0.96 | 0.007 | 0.95 | -0.13 | 0.24 | -0.02 | 0.85 | 0.09 | 0.42 | -0.08 | 0.51 | -0.14 | 0.21 |
| T | 0.20 | 0.08 | 0.06 | 0.60 | 0.12 | 0.29 | 0.04 | 0.73 | 0.03 | 0.77 | 0.003 | 0.98 | 0.02 | 0.88 | -0.08 | 0.48 |
| IN | 0.18 | 0.11 | -0.14 | 0.22 | -0.12 | 0.29 | -0.004 | 0.97 | 0.15 | 0.20 | 0.05 | 0.69 | -0.10 | 0.41 | -0.22 | 0.06 |
| I | 0.12 | 0.29 | -0.21 | 0.066 | -0.19 | 0.09 | -0.006 | 0.96 | 0.18 | 0.12 | 0.05 | 0.67 | -0.11 | 0.35 | -0.28 | **0.01** |
| IT | 0.19 | 0.10 | -0.20 | 0.076 | -0.17 | 0.14 | 0.02 | 0.85 | 0.12 | 0.28 | 0.10 | 0.40 | -0.38 | **0.001** | -0.49 | **<0.001** |
| **Subregion DVD, %** |  |  |  |  |  |  |  |  |  |  |  |  |  |  |  |  |
| Whole | 0.14 | 0.21 | -0.17 | 0.13 | -0.13 | 0.26 | -0.003 | 0.98 | 0.14 | -0.03 | 0.82 | 0.06 | 0.58 | -0.03 | 0.77 | -0.18 |
| SN | 0.11 | 0.33 | -0.13 | 0.25 | -0.06 | 0.62 | 0.06 | 0.62 | -0.04 | 0.73 | 0.16 | 0.16 | -0.13 | 0.27 | -0.15 | 0.18 |
| S | 0.11 | 0.33 | -0.08 | 0.46 | -0.03 | 0.80 | -0.04 | 0.71 | -0.11 | 0.32 | 0.07 | 0.53 | 0.04 | 0.737 | -0.06 | 0.62 |
| ST | 0.13 | 0.25 | -0.07 | 0.55 | -0.06 | 0.58 | -0.10 | 0.40 | 0.04 | 0.72 | 0.06 | 0.59 | 0.11 | 0.34 | -0.014 | 0.91 |
| N | 0.10 | 0.37 | -0.17 | 0.13 | 0.002 | 0.99 | 0.16 | 0.15 | -0.11 | 0.33 | 0.06 | 0.59 | -0.06 | 0.58 | -0.17 | 0.14 |
| C | -0.02 | 0.87 | -0.10 | 0.36 | -0.03 | 0.78 | 0.03 | 0.78 | -0.16 | 0.16 | 0.05 | 0.66 | 0.14 | 0.22 | 0.021 | 0.86 |
| T | 0.10 | 0.37 | -0.09 | 0.44 | -0.004 | 0.97 | 0.03 | 0.82 | -0.11 | 0.32 | -0.007 | 0.95 | 0.07 | 0.56 | -0.10 | 0.37 |
| IN | 0.16 | 0.17 | -0.24 | **0.04** | -0.19 | 0.09 | 0.03 | 0.79 | -0.01 | 0.94 | -0.006 | 0.96 | -0.05 | 0.65 | -0.17 | 0.13 |
| I | 0.12 | 0.31 | -0.25 | **0.04** | -0.19 | 0.10 | -0.05 | 0.67 | -0.04 | 0.76 | -0.07 | 0.54 | -0.006 | 0.96 | -0.18 | 0.11 |
| IT | 0.12 | 0.29 | -0.24 | **0.04** | -0.18 | 0.12 | 0.001 | 0.99 | 0.04 | 0.74 | 0.04 | 0.76 | -0.15 | 0.20 | -0.32 | **0.005** |

CRP: C-reactive protein; DVD: Deep vessel density; ESR: Erythrocyte sedimentation rate; FAZ: Fovea avascular zone; FD-300: vessel density of the 300µm range around FAZ; HCQ: Hydroxychloroquine; SLE: Systemic lupus erythematosus; SLEDAI-2K: Systemic lupus erythematosus disease activity index-2000; SVD: Superficial vessel density; SN: Superior nasal; S: Superior; ST: Superior temporal; N: Nasal; C: Central; T: Temporal; IN: Inferior nasal; I: Inferior; IT: Inferior temporal.

*Represented the results by Pearson correlation analysis, and the remaining were results by Spearman correlation analysis.

Supplementary file 8. Comparison of retinal vessel density differences in SLE patients with or without positive aPLs/LA, reduced complement levels, and different organs’ involvement.

|  | aPLs/LA | |  | Complement level | |  | Renal involvement | |  | NPSLE | |  | Cardiovascular involvement | |  | Hematological involvement | |  |
| --- | --- | --- | --- | --- | --- | --- | --- | --- | --- | --- | --- | --- | --- | --- | --- | --- | --- | --- |
|  | Negative（n=49） | Positive  （n=29） | P | Normal （n=31） | Decreased（n=47） | P | Absence（n=29) | Presence (n=49) | P | Absence（n=52) | Presence(n=26) | P | Absence（n=65） | Presence（n=13） | P | Absence（n=33） | Presence（n=45） | P |
| **FAZ related parameters** |  |  |  |  |  |  |  |  |  |  |  |  |  |  |  |  |  |  |
| Area, mm2 | 0.26(0.16) | 0.3(0.19) | 0.38 | 0.27(0.19) | 0.25(0.16) | 0.18 | 0.30(0.18) | 0.26(0.15) | 0.80 | 0.28(0.16)* | 0.28(0.15) | 0.49 | 0.27(0.17) | 0.26(0.13)* | 0.75 | 0.28(0.12)* | 0.25(0.17) | 0.62 |
| Perimeter, mm | 2.19(0.86) | 2.37(0.81) | 0.37 | 2.3(0.71) | 2.23(0.83) | 0.44 | 2.31(0.78) | 2.23(0.88) | 0.48 | 2.20(0.83) | 2.50(0.89) | 0.08 | 2.29(0.89) | 2.10(0.55)* | 0.31 | 2.32(0.59)* | 2.18(0.89) | 0.80 |
| Acircularity | 0.67(0.23) | 0.61(0.18)* | 0.91 | 0.63(0.14)* | 0.67(0.31) | 0.83 | 0.59(0.17) | 0.67(0.20) | 0.29 | 0.69(0.21) | 0.54(0.18)* | **0.004** | 0.61(0.29) | 0.69(0.10)* | 0.13 | 0.64(0.14)* | 0.60(0.18)* | 0.35 |
| FD-300, % | 14.64(5.72)* | 17.16(5.81)* | 0.07 | 15.26(6.36)* | 15.79(5.54)* | 0.70 | 15.65(6.12)* | 15.53(5.75)* | 0.93 | 15.55(5.48)* | 15.63(6.63)* | 0.95 | 15.68(5.89)* | 15.09(5.83)* | 0.74 | 15.34(5.47)* | 15.75(6.16)* | 0.76 |
| **Fovea SVD, %** | 9.10(3.89)* | 9.34(4.29)* | 0.80 | 8.58(3.79)* | 9.00（6.00） | 0.28 | 8.72(4.13)* | 9.47(3.96)* | 0.43 | 9.52(3.82)* | 8.54(4.38)* | 0.31 | 9.12(4.05)* | 9.54(4.01)* | 0.74 | 8.91(4.05)* | 9.40(4.03)* | 0.60 |
| **Parafovea SVD, %** |  |  |  |  |  |  |  |  |  |  |  |  |  |  |  |  |  |  |
| Temporal | 22.49(4.39)* | 24.00(6.00) | 0.52 | 21.87(4.31)* | 24.00(7.00) | 0.23 | 21.72(4.04)* | 24.00(6.00) | 0.17 | 23.00(4.26)* | 21.88(4.73)* | 0.30 | 22.02(4.22)* | 25.69(4.29)* | **0.005** | 22.79(4.29)* | 22.51(4.59)* | 0.79 |
| Superior | 26.96(5.37)* | 26.52(4.96)* | 0.72 | 26.81(5.03)* | 26.79(5.35)* | 0.99 | 25.79(5.02)* | 27.39(5.25)* | 0.19 | 27.29(5.55)* | 25.81(4.32)* | 0.24 | 26.35(5.17)* | 29.00(4.88)* | 0.09 | 26.61(5.56)* | 26.93(4.96)* | 0.79 |
| Nasal | 21.31(4.40)* | 23.00(8.00) | 0.26 | 21.74(4.30)* | 21.55(5.46)* | 0.87 | 21.86(5.30)* | 21.49(4.86)* | 0.75 | 21.81(5.09)* | 21.27(4.89)* | 0.66 | 21.48(4.79)* | 22.38(6.10)* | 0.55 | 21.79(4.78)* | 21.51(5.20)* | 0.81 |
| Inferior | 28.00(6.00) | 28.00(4.00) | 0.72 | 28.00(4.00) | 27.87(4.27) | 0.36 | 26.41(5.80)* | 28.00(4.00) | 0.25 | 28.00(4.00) | 27.54(5.66)* | 0.87 | 28.00(5.00) | 29.23(4.07)* | 0.14 | 27.30(4.00)* | 28.00(6.00) | 0.78 |
| **Perifovea SVD, %** |  |  |  |  |  |  |  |  |  |  |  |  |  |  |  |  |  |  |
| Temporal | 29.31(4.65)* | 29.38(5.29)* | 0.95 | 29.29(4.14)* | 30.00(7.00) | 0.77 | 28.62(5.40)* | 29.76(4.53)* | 0.32 | 29.85(4.87)* | 28.31(4.79)* | 0.19 | 29.92(4.88)* | 31.38(4.39)* | 0.009 | 31.00(8.00) | 29.2(4.54)* | 0.48 |
| Superior | 33.00(5.00) | 35.00(7.00) | 0.35 | 33.00(4.00) | 35.00(6.00) | 0.27 | 34.00(6.00) | 33.00(7.00) | 0.51 | 35.00(6.00) | 32.50(5.00) | **0.07** | 33.00(7.00) | 36.00(3.16) | **0.03** | 35.00(8.00) | 34.00(5.00) | 0.98 |
| Nasal | 35.00(6.00) | 35.00(7.00) | 0.91 | 33.42(4.27)* | 35.00(6.00) | 0.72 | 33.93(3.84)* | 35.00(6.00) | 0.97 | 35.00(5.00) | 32.58(5.59)* | 0.38 | 33.12(4.96)* | 36.00(2.00) | 0.06 | 35.00(6.00) | 35.00(5.00) | 0.60 |
| Inferior | 37.00(7.00) | 36.41(5.26)* | 0.75 | 38.00(7.00) | 37.00(7.00) | 0.65 | 35.62(5.70)* | 38.00(7.00) | 0.64 | 38.00(7.00) | 34.77(6.20)* | 0.14 | 37.00(7) | 38.92(3.40)* | **0.04** | 38.00(7.00) | 37.00(8.00) | 0.92 |
| **Fovea DVD, %** | 21.00(8.00) | 21.00(10.00) | 0.33 | 21.00(8.00) | 21.00(10.00) | 0.95 | 22.00(9.00) | 20.00(8.00) | 0.07 | 20.00(8.00) | 21.50(9.00) | 0.41 | 21.00(8.00) | 17.31(8.19)* | 0.18 | 21.00(8.00) | 21.00(10.00) | 0.51 |
| **Parafovea DVD, %** |  |  |  |  |  |  |  |  |  |  |  |  |  |  |  |  |  |  |
| Temporal | 29.00(12.00) | 30.00(14.00) | 0.74 | 29.00(13.00) | 29.00(12.00) | 0.98 | 30.00(11.00) | 29.00(13.00) | 0.49 | 29.00(12.00) | 29.50(13.00) | 0.85 | 29.00(12.00) | 24.15(9.67)* | 0.23 | 29.00(13.00) | 29.00(13.00) | 0.76 |
| Superior | 29.00(13.00) | 29.00(12.00) | 0.36 | 29.00(13.00) | 29.00(12.00) | 0.84 | 29.00(8.00) | 29.00(14.00) | 0.76 | 29.00(13.00) | 29.00(13.00) | 0.64 | 29.00(11.00) | 23.62(9.96)* | 0.07 | 29.00(12.00) | 30.00(13.00) | 0.27 |
| Nasal | 29.00(12.00) | 29.00(15.00) | 0.60 | 29.00(13.00) | 29.00(12.00) | 0.93 | 30.00(12.00) | 29.00(14.00) | 0.30 | 29.00(13.00) | 29.50(12.00) | 0.89 | 29.00(11.00) | 30.00(17.00) | 0.60 | 30.00(13.00) | 29.00(13.00) | 0.53 |
| Inferior | 29.00(12.00) | 30.00(14.00) | 0.83 | 29.00(13.00) | 29.00(12.00) | 0.35 | 29.00(11.00) | 29.00(14.00) | 0.75 | 29.00(12.00) | 30.00(13.00) | 0.43 | 29.00(12.00) | 29.00(16.00) | 0.86 | 29.00(12.00) | 29.00(13.00) | 0.62 |
| **Perifovea DVD, %** |  |  |  |  |  |  |  |  |  |  |  |  |  |  |  |  |  |  |
| Temporal | 29.00(11.00) | 30.00(12.00) | 0.36 | 29.00(11.00) | 29.00(11.00) | 0.89 | 30.00(7.00) | 29.00(11.00) | 0.41 | 29.00(11.00) | 29.00(11.00) | 0.75 | 29.00(10.00) | 24.46(9.12)* | 0.62 | 29.00(11.00) | 29.00(12.00) | 0.41 |
| Superior | 29.00(12.00) | 29.00(13.00) | 0.64 | 29.00(12.00) | 29.00(12.00) | 0.85 | 29.00(11.00) | 29.00(13.00) | 0.81 | 29.00(12.00) | 28.50(12.00) | 0.53 | 29.00(11.00) | 23.92(9.44)* | 0.26 | 28.00(11.00) | 29.00(13.00) | 0.98 |
| Nasal | 29.00(12.00) | 29.00(13.00) | 0.70 | 29.00(12.00) | 29.00(13.00) | 0.88 | 29.00(9.00) | 29.00(13.00) | 0.46 | 29.00(12.00) | 29.00(13.00) | 0.58 | 29.00(10.00) | 28.00(15.00) | 0.49 | 29.00(13.00) | 29.00(13.00) | 0.53 |
| Inferior | 29.00(11.00) | 29.00(13.00) | 0.79 | 29.00(12.00) | 29.00(12.00) | 0.89 | 29.00(7.00) | 29.00(13.00) | 0.39 | 29.00(12.00) | 29.00(11.00) | 0.92 | 29.00(9.00) | 23.85(9.09)* | 0.25 | 28.00(11.00) | 29.00(13.00) | 0.45 |
| **Subregion SVD,%** |  |  |  |  |  |  |  |  |  |  |  |  |  |  |  |  |  |  |
| Whole | 24.35(3.25)* | 25.31(3.18)* | 0.21 | 23.71(2.90)* | 24.36(3.31)* | **0.03** | 24.85±3.18* | 24.62(3.30) | 0.76 | 25.21(3.16)* | 23.70(3.22) | 0.052 | 24.33(3.26) | 26.59(2.43) | **0.02** | 24.88(3.13)* | 24.68(3.34)* | 0.68 |
| SN | 22.43(5.96)* | 24.00(13.00) | 0.57 | 20.32(6.21)* | 24.00(11.00） | **0.01** | 23.55(5.53)* | 22.18(7.01)* | 0.37 | 23.00(12.00) | 21.96(6.37)* | 0.54 | 22.86(6.29)* | 21.85(7.68)* | 0.61 | 22.91(7.12)* | 22.53(6.08)* | 0.80 |
| S | 30.00(6.00) | 29.69(3.76)* | 0.86 | 28.87(4.34)* | 29.62(4.49)* | 0.47 | 29.76(3.45)* | 29.06(4.92)* | 0.50 | 30.50(6.00) | 29.00(6.00) | 0.12 | 30.00(6.00) | 31.85(3.00)* | **0.02** | 30.33(4.11)* | 30.00(7.00) | 0.11 |
| ST | 21.02(6.75)* | 22.79(6.32)* | 0.26 | 22.90(5.82)* | 20.87(7.03)* | 0.19 | 23.07(5.69)* | 20.86(7.03)* | 0.16 | 23.50(9.00) | 19.92(6.40)* | 0.057 | 21.23(6.64)* | 23.92(6.21)* | 0.18 | 23.00(8.00) | 20.87(6.44)* | 0.16 |
| N | 29.43(5.40)* | 29.34(6.77)* | 0.95 | 28.00(9.00) | 32.00(8.00) | **0.003** | 30.28(4.68)* | 28.88(6.51)* | 0.32 | 29.77(6.15)* | 28.65(5.41)* | 0.44 | 29.58(5.94)* | 28.46(5.85)* | 0.54 | 29.88(9.00)* | 29.04(6.34)* | 0.54 |
| C | 34.00(5.00) | 33.10(3.85)* | 0.74 | 32.77(3.42)* | 34.00(6.00) | 0.57 | 34.00(7.00) | 34.00(4.00) | 0.96 | 34.00(5.00) | 31.92(4.16)* | 0.10 | 34.00(5.00) | 35.38(2.47)* | **0.005** | 34.00(6.00) | 34.00(4.00) | 0.58 |
| T | 25.33(4.85)* | 26.24(5.24)* | 0.44 | 26.06(4.12)* | 25.00(11.00) | 0.61 | 25.97(4.79)* | 25.49(5.14)* | 0.69 | 26.08(5.17)* | 24.85(4.58)* | 0.31 | 25.28(9.00)* | 27.62(6.16)* | 0.12 | 26.52(4.97)* | 25.04(4.96)* | 0.20 |
| IN | 18.00(7.00) | 20.34(4.19)* | 0.18 | 18.03(5.46)* | 19.83(4.82)* | 0.13 | 18.48(5.34)* | 19.49(5.02)* | 0.41 | 21.00(6.00) | 18.00(4.96)* | 0.07 | 18.69(5.17)* | 21.23(4.53)* | 0.10 | 18.42(4.94)* | 19.62(5.26）* | 0.31 |
| I | 22.96(6.12)* | 26.00(10.00) | 0.12 | 22.10(6.7)* | 24.72(5.48)* | 0.06 | 25.00(12.00) | 24.41(5.43)* | 0.32 | 26.00(9.00) | 22.54(5.78)* | 0.15 | 24.00(10.00) | 28.00(2.71)* | **0.004** | 22.94(5.86)* | 26.00(11.00) | 0.24 |
| IT | 17.63(6.05)* | 19.00(11.00) | 0.65 | 15.29(5.09)* | 21.00(9.00) | **0.001** | 17.34(5.46)* | 18.14(6.07)* | 0.56 | 18.10(5.88)* | 17.35(5.80)* | 0.60 | 17.00(9.00) | 21.00(3.37)* | **0.03** | 17.15(8.00)* | 20.00(11.00) | 0.32 |
| **Subregion DVD, %** |  |  |  |  |  |  |  |  |  |  |  |  |  |  |  |  |  |  |
| Whole | 27.11(7.22) | 27.56(9.06) | 0.32 | 26.11(8.44) | 27.56(7.56) | 0.06 | 27.44(5.89) | 27.11(8.89) | 0.69 | 27.28(7.94) | 27.22(8.94) | 0.464 | 27.11(6.67) | 26.05(6.52)* | 0.32 | 27.56(8.22) | 27.11(8.00) | 0.53 |
| SN | 27.00(9.00) | 27.00(9.00) | 0.87 | 23.35(5.39)* | 28.00(8.00) | **0.02** | 28.00(8.00) | 26.00(9.00) | 0.36 | 27.00(9.00) | 27.00(8.00) | 0.38 | 27.00(9.00) | 24.85(7.14)* | 0.67 | 26.00(10.00) | 27.00(9.00) | 0.88 |
| S | 29.00(8.00) | 29.00(9.00) | 0.71 | 28.00(9.00) | 29.00(7.00) | 0.36 | 29.00(7.00) | 29.00(9.00) | 0.92 | 29.00(8.00) | 29.00(9.00) | 0.32 | 29.00(8.00) | 27.31(6.99)* | 0.42 | 29.00(8.00) | 28.00(9.00) | 0.19 |
| ST | 27.00(8.00) | 28.00(10.00) | 0.58 | 28.00(9.00) | 27.00(8.00) | 0.91 | 28.00(8.00) | 27.00(9.00) | 0.50 | 28.00(9.00) | 25.12(5.23)* | 0.39 | 28.00(8.00) | 27.23(6.92)* | 0.31 | 29.00(10.00) | 27.00(8.00) | 0.40 |
| N | 29.00(8.00) | 29.00(9.00) | 0.90 | 28.00(8.00) | 30.00(7.00) | **0.02** | 29.00(7.00) | 28.00(9.00) | 0.89 | 29.00(9.00) | 28.00(8.00) | 0.66 | 29.00(7.00) | 26.15(6.95)* | 0.65 | 29.00(7.00) | 29.00(9.00) | 0.89 |
| C | 29.00(10.00) | 30.00(12.00) | 0.18 | 29.00(11.00) | 29.00(10.00) | 0.77 | 29.00(7.00) | 29.00(12.00) | 0.96 | 29.00(11.00) | 29.00(11.00) | 0.89 | 29.00(10.00) | 26.23(7.83)* | 0.45 | 29.00(10.00) | 29.00(12.00) | 0.86 |
| T | 29.00(7.00) | 30.00(9.00) | 0.36 | 28.00(9.00) | 29.00(5.00) | 0.41 | 29.00(6.00) | 28.00(8.00) | 0.68 | 29.00(8.00) | 29.00(10.00) | 0.60 | 29.00(6.00) | 27.54(7.17)* | 0.46 | 29.00(8.00) | 29.00(9.00) | 0.39 |
| IN | 24.00(9.00) | 25.00(9.00) | 0.21 | 24.00(8.00） | 26.00(6.00) | 0.09 | 24.00(8.00) | 26.00(8.00) | 0.70 | 25.00(8.00) | 24.00(10.00) | 0.37 | 25.00(8.00) | 24.62(5.53)* | 0.23 | 25.00(8.00) | 25.00(8.00) | 0.75 |
| I | 25.00(7.00) | 26.00(9.00) | 0.23 | 25.00(8.00） | 26.00(7.00) | 0.06 | 25.00(7.00) | 26.00(8.00) | 0.42 | 25.50(8.00) | 25.00(9.00) | 0.63 | 25.00(8.00) | 26.00(6.23)* | 0.17 | 25.00(8.00) | 25.00(8.00) | 0.79 |
| IT | 22.65(5.11)* | 23.00(8.00) | 0.66 | 20.94(4.75)* | 25.00(7.00) | **0.01** | 24.00(7.00) | 22.65(4.95)* | 0.51 | 22.85(4.73)* | 24.00(9.00) | 0.92 | 22.35(4.83)* | 24.54(5.43)* | 0.15 | 22.48(4.75)* | 22.89(5.16)* | 0.73 |

aPLS: Antiphospholipid antibodies; DVD: Deep vessel density; FAZ: Fovea avascular zone; FD-300: vessel density of the 300µm range around FAZ; HCQ: Hydroxychloroquine; LA: Lupus anticoagulant; NPSLE: Neuropsychiatric lupus; SLE: Systemic lupus erythematosus; SLEDAI-2K: Systemic lupus erythematosus disease activity index-2000; SVD: Superficial vessel density; SN: Superior nasal; S: Superior; ST: Superior temporal; N: Nasal; C: Central; T: Temporal; IN: Inferior nasal; I: Inferior; IT: Inferior temporal.

Supplementary file 9. Clinical characteristics of patients with different disease activity in SLE patients without retinopathy.

|  | Disease activity | | |  |
| --- | --- | --- | --- | --- |
|  | Mild(n=18) | Moderate(n=31) | Severe(n=29) | P |
| Age (years) | 39.50(14.94)* | 39.50(11.78)* | 33.21(12.39)* | 0.11 |
| Gender (female), n(%) | 15(83.30) | 27(87.10) | 23(79.30) | 0.69 |
| aPLs or LA, n(%) | 8(44.40) | 8(25.80) | 13(44.80) | 0.23 |
| Anti-Smith antibody, n (%) | 3(16.70) | 5(16.10) | 10(34.50) | 0.20 |
| Anti-dsDNA antibody, n (%) | 6(33.30) | 18(58.10) | 16(55.20) | 0.21 |
| Low complement, n (%) | 6(33.30) | 16(51.60) | 25(86.20) | **<0.001** |
| Comorbidity,n(%) |  |  |  |  |
| Hypertension | 4(22.20) | 11(35.50) | 14(48.30) | 0.18 |
| Hyperlipidemia | 3(16.70) | 12(38.70) | 7(24.10) | 0.21 |
| Diabetes mellitus | 3(16.70) | 5(16.10) | 0(00) | 0.05 |
| Coronary heart disease | 1(5.60) | 2(6.50) | 1(3.40) | 1.00 |
| ESR, mm/h | 8.00(10.00) | 13.00(24.00) | 25.00(55.00) | **0.01** |
| CRP, mg/L | 0.96(3.00) | 1.36(7.00) | 0.80(6.00) | 0.85 |
| **Involved organs, n(%)** |  |  |  |  |
| Skin | 4(22.20) | 2(6.50) | 6(20.70) | 0.19 |
| Hematological system | 13(72.20) | 18(58.10) | 14(48.30) | 0.26 |
| Neuropsychiatric system | 6(33.30) | 9(29.00) | 11(37.90) | 0.77 |
| Kidney | 8(44.40) | 18(58.10) | 23(79.30) | **0.04** |
| Polyplasma membrane cavity effusion | 0(0.00) | 3(9.70) | 8(27.60) | **0.008** |
| Lung | 7(38.90) | 6(19.40) | 6(20.70) | 0.29 |
| Cardiovascular system | 1(5.60) | 4(12.90) | 8(27.60) | 0.14 |
| Secondary APS | 4(22.20) | 6(19.40) | 6(20.70) | 1.00 |
| **Treatment** |  |  |  |  |
| Glucocorticoids, n (%) | 18(100) | 31(100) | 29(100) | / |
| HCQ, n(%) | 5.50(6.00) | 2(4.00) | 1(4.00) | **0.01** |
| Azathioprine, n (%) | 1(5.60) | 3(9.70) | 2(6.90) | 1.00 |
| Mycophenolate mofetil, n (%) | 10(55.60) | 18(58.10) | 14(48.30) | 0.74 |
| Cyclophosphamide, n (%) | 10(55.60) | 14(45.20) | 20(69.00) | 0.17 |
| Tacrolimus, n (%) | 7(38.90) | 14(45.20) | 6(20.70) | 0.12 |
| Ciclosporin,n(%) | 2(11.10) | 2(6.50) | 0(0.00) | 0.22 |
| Rituximab, and n (%) | 1(5.60) | 3(9.70) | 1(3.40) | 0.84 |
| Belizumab, n (%) | 2(11.10) | 3(9.70) | 5(17.20) | 0.69 |
| IVIG, n(%) | 5(27.80) | 2(6.50) | 5(17.20) | 0.12 |
| **Ophthalmic conditions** |  |  |  |  |
| BCVA (logMAR) | 0.00(0.10) | 0.00(0.10) | 0.00(0.10) | 0.22 |
| IOP (mmHg) | 14.44(2.04）* | 15.53(2.32)* | 13.30(4.10) | 0.09 |
| SE | 0.00(1.15) | 0.00(2.88) | 0.00(2.25) | 0.89 |

APLs: Anti-phospholipid antibodies; BCVA: Best corrected visual acuity; CRP: C-reactive protein; ESR: Erythrocyte sedimentation rate; HCQ: Hydroxychloroquine; IOP: Intraocular pressure; IVIG: Intravenous immunoglobulin; LA: lupus anticoagulant; SE: Spherical equivalent; SLE: Systemic lupus erythematosus.

*Represented the mean (standard deviation), and the remaining quantitative data were the median (interquartile range).

Supplementary file 10 Comparison of retinal vessel density between SLE patients with different disease activity and control group.

|  | SLE patients without retinopathy | | | Control (n=65) | P |
| --- | --- | --- | --- | --- | --- |
|  | Mild（n=18) | Moderate(n=31) | Severe(n=29) |  |  |
| **FAZ related parameters** |  |  |  |  |  |
| Area, mm2 | 0.22(0.21) | 0.29(0.17)* | 0.27(0.15) | 0.27(0.25) | 0.77 |
| Perimeter, mm | 2.12(1.00) | 2.31(0.90) | 2.30(0.77) | 2.35(1.05) | 0.71 |
| Acircularity | 0.63(0.14)* | 0.62(0.16)* | 0.66(0.27) | 0.68(0.20) | 0.99 |
| FD-300, % | 15.01(7.72)* | 14.19(5.42)* | 17.41(4.54)* | 13.51(5.94)* | **0.03** |
| **Fovea SVD, %** | 9.00(4.17)* | 8.52(3.632)* | 10.03(4.29)* | 7.22(4.40)* | **0.02** |
| **Parafovea SVD, %** |  |  |  |  |  |
| Temporal | 22.22(4.52)* | 21.52(3.39)* | 24.07(5.05)* | 19.72(5.10)* | **<0.001** |
| Superior | 25.94(5.50)* | 26.29(4.38)* | 27.86(5.77)* | 25.17(5.07)* | 0.14 |
| Nasal | 22.78(5.25)* | 20.81(4.09)* | 21.79(5.70)* | 19.00(6.00) | **0.004** |
| Inferior | 28.06(5.99)* | 27.00(5.00) | 28.14(4.70)* | 26.00(8.00) | **0.04** |
| **Perifovea SVD, %** |  |  |  |  |  |
| Temporal | 29.22(4.61)* | 29.00(8.00) | 31.00(8.00) | 28.00(6.00) | **0.005** |
| Superior | 33.50(5.00) | 31.90(4.32)* | 35.00(6.00) | 33.55(4.93)* | 0.15 |
| Nasal | 33.89(4.91)* | 33.55(3.50)* | 35.00(8.00) | 32.00(6.00) | **0.04** |
| Inferior | 38.50(7.00) | 36.29(4.28)* | 38.00(8.00) | 33.72(5.03)* | **0.009** |
| **Fovea DVD, %** | 22.00(5.00) | 21.00(10.00) | 18.00(6.69)* | 18.00(12.00) | 0.18 |
| **Parafovea DVD, %** |  |  |  |  |  |
| Temporal | 31.00(3.00) | 29.00(13.00) | 28.00(13.00) | 29.00(13.00) | 0.15 |
| Superior | 30.00(2.00) | 29.00(13.00) | 28.00(14.00) | 28.00(12.00) | 0.40 |
| Nasal | 30.00(3.00) | 29.00(13.00) | 29.00(15.00) | 29.00(13.00) | 0.44 |
| Inferior | 29.50(3.00) | 29.00(13.00) | 29.00(14.00) | 29.00(14.00) | 0.87 |
| **Perifovea DVD, %** |  |  |  |  |  |
| Temporal | 30.00(1.00) | 29.00(12.00) | 29.00(11.00) | 29.00(13.00) | 0.31 |
| Superior | 28.50(10.00) | 29.00(13.00) | 29.00(13.00) | 28.00(12.00) | 0.43 |
| Nasal | 29.00(2.00) | 29.00(12.00) | 28.00(13.00) | 29.00(13.00) | 0.31 |
| Inferior | 29.00(3.00) | 29.00(13.00) | 28.00(13.00) | 28.00(14.00) | 0.58 |
| **Subregion SVD,%** |  |  |  |  |  |
| Whole | 23.42(3.95) | 24.90(2.23) | 25.30(3.55) | 23.76(2.43)* | **0.035** |
| SN | 21.89(6.85)* | 22.13(6.06)* | 23.79(6.80)* | 24.54(5.75)* | 0.21 |
| S | 28.67(4.24)* | 30.00(6.00) | 29.77(4.75)* | 28.92(3.75)* | 0.56 |
| ST | 20.83(6.26)* | 21.19(7.38)* | 25.00(7.00) | 21.82(5.68)* | 0.67 |
| N | 28.83(5.88)* | 29.55(6.33)* | 32.00(10.00) | 30.00(9.00) | 0.90 |
| C | 34.00(3.00) | 34.00(5.00) | 35.00(7.00) | 32.00(5.00) | **0.03** |
| T | 25.17(5.11)* | 24.81(4.74)* | 26.9(5.08)* | 23.31(4.79)* | **0.01** |
| IN | 16.06(6.25)* | 20.39(3.94)* | 19.66(4.89)* | 16.78(5.13)* | **0.001** |
| I | 21.5(6.80)* | 26（8） | 23.93(5.58)* | 23.68(6.09)* | **0.005** |
| IT | 14.78(6.15)* | 19.29(4.98)* | 18.21(5.94)* | 17(4.29) | **0.03** |
| **Subregion DVD, %** |  |  |  |  |  |
| Whole | 26.28(5.28) | 27.11(7.56) | 27.44(9.00) | 26.67(9.00) | 0.31 |
| SN | 27.50(8.00) | 26.00(9.00) | 28.00(10.00) | 27.00(10.00) | 0.58 |
| S | 28.50(5.00) | 29.00(9.00) | 29.00(10.00) | 29.00(11.00) | 0.29 |
| ST | 27.00(10.00) | 27.00(10.00) | 26.62(5.93)* | 27.00(9.00) | 0.49 |
| N | 28.00(4.00) | 29.00(9.00) | 29.00(10.00) | 28.00(9.00) | 0.22 |
| C | 29.00(2.00) | 29.00(12.00) | 29.00(11.00) | 29.00(11.00) | 0.18 |
| T | 29.00(5.00) | 28.00(9.00) | 30.00(9.00) | 27.00(9.00) | 0.10 |
| IN | 24.00(10.00) | 25.00(7.00) | 25.00(8.00) | 22.00(8.00) | 0.10 |
| I | 23.5(4.96)* | 25.00(8.00) | 26.00(9.00) | 25.00(8.00) | 0.24 |
| IT | 22.00(8.00) | 24.00(7.00) | 23.21(5.25)* | 24.00(9.00) | 0.53 |

DVD: Deep vessel density; FAZ: Fovea avascular zone; FD-300: vessel density of the 300µm range around FAZ; SLE: Systemic lupus erythematosus; SVD: Superficial density; SN: Superior nasal; S: Superior; ST: Superior temporal; N: Nasal; C: Central; T: Temporal; IN: Inferior nasal; I: Inferior; IT: Inferior temporal.

*Represented the mean (standard deviation), and the remaining quantitative data were the median (interquartile range ).

Supplementary file 11. Clinical characteristics of patients in OCTA studies regarding SLE without retinopathy patients.

| Author | Publication year | Age^*^ | Patients/Control | BCVA^*^ (logMAR) | Duration of SLE^*^ | SLEDA-2K(Mean）/Disease activity group (No. patients) |
| --- | --- | --- | --- | --- | --- | --- |
| Conigliaro et al | 2018 | 49.60 (13.60) | 26(52 eyes) /20(40 eyes) | 0.00(0.10) | 15.10(7.70) | 4.30 |
| Bao et al | 2019 | 34.90 (11.80) | 32(58eyes) /50(50eyes) | -0.01(0.04) | 3.80(3.00) | 7.70 |
| Pichi et al | 2020 | 42.30 (13.40) | 15(30eyes) /15(30eyes) | 0.10(/) | 11.30(6.80) | 0: n=6 1-8: n=8 ＞8: n=1 |
| Arfeen et al | 2020 | 29.20 (7.90) | 20(20eyes) /20(20eyes) | 0.07(1.10) | / | No activity: n=8 Mild: n=2 Moderate: n=10 |
| Shi et al | 2021 | 33.75 (9.08) | 12(24eyes) /12(24eyes) | 0.12(0.53) | 4.33(2.56) | 4.25 |
| Liu et al | 2021 | 33.80 (9.49) | 12(24eyes) /12(24eyes) | 0.17(0.20) | 4.33(2.67) | 4.25 |
| Subasi et al | 2022 | 43.37 (12.05) | 60(60eyes) /60(60eyes) | 0.00(/) | 11.63(6.94) | 5.68 Mild/Moderate/Severe=42/10/8 |
| Pelegrín et al | 2022 | 45.00(/) | 78(78eyes) /80(80eyes) | 0.00(/) | 8.60(/) | ≤4: n=74 (95%) |
| Ermurat et al | 2022 | 39.00(/) | 47(47eyes) /41(41eyes) | 0.10(/) | / | ≤5: n=26 ＞6: n=21 |

BCVA: Best corrected visual acuity; OCTA: Optical coherence tomography angiography; SLE: Systemic lupus erythematosus; SLEDAI: Systemic lupus erythematosus disease activity index;

*Respresented mean (standard deviation).
